# Functional and clinical outcomes of propeller flaps in lower extremity reconstruction: A systematic review

**DOI:** 10.1016/j.jpra.2026.03.009

**Published:** 2026-03-10

**Authors:** Luigi Annacontini, Luigi Cagiano, Vincenzo Verdura, Roberta Zupo, Fabio Castellana, Domenico Parisi, Aurelio Portincasa

**Affiliations:** a Department of Plastic and Reconstructive Surgery, University of Foggia, Viale Luigi Pinto 1, 71100 Foggia, Italy; b Department of Interdisciplinary Medicine (DIM), University of Bari Aldo Moro, Piazza Giulio Cesare 11, 70100 Bari, Italy

**Keywords:** Propeller perforator flap, Lower-extremity reconstruction, Functional outcomes, Soft-tissue defects, Systematic review

## Abstract

**Background:**

Soft-tissue reconstruction of the distal leg, ankle, and foot remains challenging due to limited local tissue and compromised vascularity. Propeller perforator flaps have emerged over the past 2 decades as an alternative to free flaps, offering preservation of major vascular axes and reduced donor-site morbidity. However, clinical outcomes remain heterogeneous, and the reliability and functional benefits of PPFs are still debated.

**Methods:**

A PRISMA-2020–compliant systematic review (PROSPERO: CRD420251148124) was conducted through August 2025 across PubMed, MEDLINE, Embase, Ovid, and Web of Science. Eligible studies included observational designs, case series, and case reports on PPFs for lower-extremity reconstruction. Primary outcomes included flap survival, necrosis, venous congestion, infection, reoperation, functional recovery, and quality of life. The risk of bias was assessed using the Joanna Briggs Institute tools and the Newcastle–Ottawa Scale.

**Results:**

Fifty-one studies (*n* = 1474 patients) met the inclusion. Trauma was the leading etiology (88%), with reconstructions most often involving the ankle/malleolar and heel regions. Posterior tibial and peroneal artery perforators were used in 74% of cases. Complete flap survival was reported in 47% of studies; partial necrosis in 28%, and total flap loss in 10%. Venous congestion was common but often self-limiting. Functional outcomes were inconsistently reported and generally favorable in fewer than half of the studies. Methodological quality was moderate to good in most reports.

**Conclusions:**

Propeller perforator flaps are a feasible reconstructive option for lower limb defects, although outcomes remain variable. Further prospective, comparative studies are needed to establish their long-term reliability and functional impact.

## Introduction

Reconstruction of lower-extremity soft tissue defects remains a demanding challenge in reconstructive surgery, particularly in the distal leg, ankle, and foot, where limited local tissue, poor vascularity, and exposure of vital structures complicate management. These defects often result from trauma, infection, vascular disease, or chronic wounds, leading to prolonged disability or limb loss if inadequately treated. Reconstructive goals extend beyond coverage to restoring limb function, mechanical durability, and acceptable aesthetics to support rehabilitation and quality of life.

Traditional options—skin grafts, local flaps, and microsurgical free transfers—each have limitations: grafts lack durability, while free flaps, though reliable, require microsurgical expertise and entail donor-site morbidity. The modern evolution of lower-leg coverage with reliable local tissues was strongly influenced by the landmark fasciocutaneous flap concept introduced by Pontén.^1^ In parallel, the paradigm of early microsurgical reconstruction for complex extremity trauma was consolidated by Godina, supporting free-tissue transfer as a cornerstone approach when indicated.^2^ Local and regional perforator flaps have thus emerged as an intermediate solution, combining vascularized tissue reliability with reduced operative complexity and morbidity.^3^ Clinical experience with pedicled perforator flaps in extremity soft-tissue defects further supported this intermediate strategy, demonstrating feasibility and acceptable outcomes in early series.^4^

Propeller perforator flaps (PPFs)—island flaps based on a single perforator, rotating up to 180°—have gained traction over the past 2 decades for lower extremity reconstruction. They offer advantages like preservation of major vascular axes, reduced donor morbidity, and avoidance of complex microsurgery, with tissue matching (“like-for-like”).^3^

Despite these advantages, the adoption of propeller flaps is not without limitations and concerns. The most frequently reported complications include venous congestion, partial flap necrosis, wound dehiscence, infection, and, in rare cases, total flap loss.^5^^,^^6^ The incidence of these adverse outcomes varies widely among published series, reflecting differences in flap design, patient populations, comorbidities, and perioperative protocols. Moreover, the learning curve associated with meticulous perforator dissection and flap rotation remains an important consideration for surgeons transitioning from more conventional reconstructive approaches. Beyond flap survival, an equally important but less consistently reported domain is functional outcome, including restoration of ambulation, range of motion, pain relief, and return to daily activities. Quality of life, patient satisfaction, and long-term durability of reconstruction are also critical metrics in contemporary surgical evaluation. Yet, systematic evidence regarding these outcomes in propeller flap reconstruction remains scarce.^7^^,^^8^

Given the growing body of literature on perforator-based propeller flaps, a comprehensive synthesis of the available data is timely and necessary. Previous narrative reviews have described technical refinements and case series, but a systematic evaluation of outcomes—including survival rates, complications, healing, functional recovery, and patient-reported measures—is still lacking.^3^ Such a synthesis is essential not only for defining the current role of propeller flaps in the reconstructive algorithm of the lower extremity but also for identifying evidence gaps and future research priorities.

The present systematic review was conducted to critically evaluate the clinical and functional outcomes of propeller flaps in lower extremity reconstruction. Our objectives were to assess flap survival, complication rates, wound healing, revision surgery, functional recovery, and quality-of-life measures. By consolidating current evidence, we aim to clarify the effectiveness, safety, and limitations of propeller flaps and to define their role in the reconstructive management of lower limb soft tissue defects.

## Methods

### Study design and registration

This systematic review was conducted in accordance with the PRISMA 2020 guidelines.^9^ The protocol was prospectively registered in the International Prospective Register of Systematic Reviews (PROSPERO; registration number CRD420251148124).

### Literature search strategy

**Figure 1:**
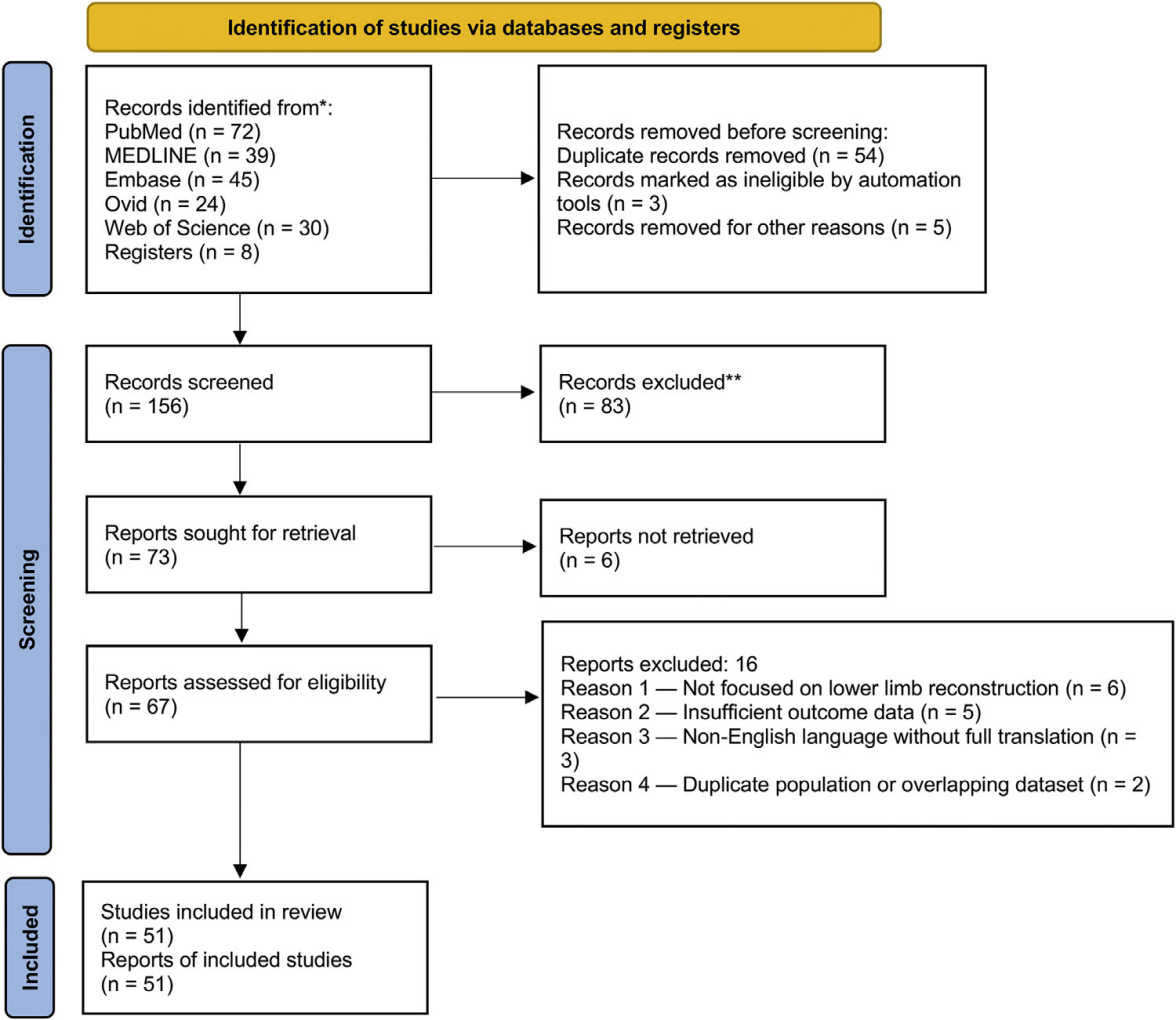
PRISMA flow diagram. ^⁎^ Consider, if feasible to do so, reporting the number of records identified from each database or register searched (rather than the total number across all databases/registers). ^⁎⁎^ If automation tools were used, indicate how many records were excluded by a human and how many were excluded by automation tools.

A comprehensive literature search was performed across PubMed, MEDLINE, Embase, Ovid, and Web of Science, covering all studies published up to 30 August 2025, with no language restrictions. The search strategy combined Medical Subject Headings (MeSH) and free-text terms across four main concept blocks: (i) propeller/perforator flaps (“Propeller Flap”, “Propeller Flaps”, “Pedicled Perforator Flap”, “Perforator Flap[s]”), (ii) anatomical location (“Lower Extremity”, “Leg”, “Lower Limb”, “Distal Leg”, “Foot”, “Ankle”), (iii) indication (“Reconstructive Surgical Procedures”, “Soft Tissue Defects”, “Reconstruction”), and (iv) outcomes (“Treatment Outcome”, “Complications”, “Flap Survival”, “Necrosis”, “Venous Congestion”, “Reoperation”, “Functional Outcome”, “Quality of Life”). Database-specific syntax, Boolean operators, truncation, and proximity operators were applied as appropriate. To maximize sensitivity, no filters for study design were applied at the search stage. The reference lists of all included articles and relevant reviews were hand-searched, and forward citation tracking was conducted to identify additional studies. Duplicate records were removed before the screening process. The full search strategy is detailed in Table 1, and the study selection process is summarized in Figure 1 (PRISMA flow diagram).

**Table 1:** PICO framework for the literature search strategy.

Item	Definition	Search terms
P – Population	Patients with soft tissue defects of the distal lower extremity (leg, ankle, foot)	“Lower Extremity”[Mesh], “Lower extremity”[tiab], “Leg”[tiab], “Leg Injuries”[Mesh], “Lower Limb”[tiab], “Distal Leg”[tiab], “Foot”[tiab], “Ankle”[tiab], “Soft Tissue Defects”[Mesh], “Soft tissue defect*”[tiab]
I – Intervention	Surgical reconstruction using propeller flaps or pedicled perforator flaps	“Propeller Flap”[tiab], “Propeller Flaps”[tiab], “Pedicled Perforator Flap”[tiab], “Perforator Flap”[tiab], “Perforator Flaps”[Mesh]
C – Comparison	Not explicitly stated; may include other reconstructive techniques (e.g., free flaps, local flaps, skin grafts) or no comparison (single-arm outcome studies)	–
O – Outcomes	Surgical and clinical outcomes: flap survival, necrosis, venous congestion, complications, infection, wound healing, dehiscence, reoperation, revision surgery, functional recovery, quality of life, success/failure rates	“Treatment Outcome”[Mesh], “Surgical Outcome”[tiab], “Clinical Outcome”[tiab], “Complications”[Mesh], “Flap Survival”[tiab], “Flap Necrosis”[tiab], “Venous Congestion”[tiab], “Success Rate”[tiab], “Wound Healing”[Mesh], “Healing”[tiab], “Infection”[Mesh], “Surgical Revision”[tiab], “Revision Surgery”[tiab], “Dehiscence”[tiab], “Reoperation”[tiab], “Functional Outcome”[tiab], “Quality of Life”[Mesh], “Graft Survival”[tiab], “Failure”[tiab]
Exclusion keywords	To exclude reviews and secondary research	“review”[tiab], “meta-analysis”[tiab], “systematic review”[tiab]

### Study selection

Two reviewers (FC, RZ) independently screened titles and abstracts for relevance. Full-texts of potentially eligible studies were then assessed for inclusion according to predefined criteria. Disagreements were resolved through discussion or by consultation with a third reviewer (LA) in case of persistent disagreement.

### Eligibility criteria (PICO framework)

• **Population (P):** Patients undergoing reconstruction of soft tissue defects of the lower extremity (leg, ankle, foot) with propeller or pedicled perforator flaps. Both acute and chronic defects were considered, regardless of etiology (trauma, oncologic, infection, chronic ulcers).
• **Intervention (I):** Use of propeller perforator flaps (PPFs) for defect coverage.
• **Comparator (C):** Alternative reconstructive techniques (e.g., free flaps, local flaps, skin grafts) or absence of comparator.
• **Outcomes (O):** Clinical outcomes including flap survival, partial/total necrosis, venous congestion, wound healing, infection, wound dehiscence, reoperation, functional recovery (e.g., ambulation, range of motion), and patient-reported outcomes such as quality of life.

Eligible study designs included randomized controlled trials, prospective and retrospective cohort studies, case–control studies, case series, and single-case reports. Although case reports typically provide low-level evidence, they were included to maximize comprehensiveness and to capture rare complications or innovative technical modifications.

### Quality assessment

Given the study mix (25/51 case series, 17/51 cohorts, 8/51 case reports, and one cross-sectional), design-specific appraisal tools were applied and judgments harmonized across designs. Case series and reports were evaluated with the JBI checklist, focusing on inclusion clarity, recruitment completeness, participant characterization (demographics, defect etiology, comorbidities), definition of intervention (propeller flap type, perforator source, rotation arc, adjuncts), standardized outcome measures (flap survival, venous patency, functional scores, PROMs), follow-up adequacy, and transparency in reporting adverse events and reoperations.

Cohort studies were assessed using the Newcastle–Ottawa Scale and NIH tool, covering representativeness, comparability, and objective outcome assessment. Key confounders included defect size, location, etiology, infection, diabetes, vascular status, timing, fixation method, and surgeon/center effects. Follow-up was typically ≥6–12 months, with acceptable attrition and clear definitions for flap loss and complications. The single cross-sectional study was appraised with JBI/AXIS for sampling adequacy, validity of measures, and bias handling.

Two reviewers (FC, RZ) independently performed the quality assessment. Any discrepancies were resolved through discussion and, if necessary, by consultation with a third reviewer (LA). The overall certainty of the evidence for each outcome was further graded using the GRADE approach,^10^ categorizing the strength of evidence as high, moderate, low, or very low.

## Results

### Study selection and characteristics

A total of 51 studies^5^^,^^8^^,^^11^, ^12^, ^13^, ^14^, ^15^, ^16^, ^17^, ^18^, ^19^, ^20^, ^21^, ^22^, ^23^, ^24^, ^25^, ^26^, ^27^, ^28^, ^29^, ^30^, ^31^, ^32^, ^33^, ^34^, ^35^, ^36^, ^37^, ^38^, ^39^, ^40^, ^41^, ^42^, ^43^, ^44^, ^45^, ^46^, ^47^, ^48^, ^49^, ^50^, ^51^, ^52^, ^53^, ^54^, ^55^ met the inclusion criteria (Table 2, Table 3), detailing design, country, sample size, intervention type, outcomes (flap survival, venous patency, swelling, functional and radiological results, complications, PROMs), and main findings. Publications ranged from 2007 to 2025, with a median year of 2020 (IQR 2016–2022), showing a clear increase in recent years.

**Table 2:** Characteristics of included studies (*n* = 51). Perforator-based propeller flaps for distal lower-limb reconstruction.

First author, Year	Design	Country	N	Age	Sex	Etiology of defects	Defect locations	Intervention (Flap type)	Rotation arc	Follow-up	Main findings
Chen G 2025	Prospective case series	China	15	65–90 y	6 M/9 F	Trauma (*n* = 9) Diabetic foot (*n* = 3) Infection (*n* = 3)	Anterior ankle (*n* = 3) Medial malleolus (*n* = 5) Posterior ankle/heel (*n* = 7)	Spiral propeller perforator flap with superficial vein anastomosis	120–180°	12 months	Superficial-vein–anastomosed spiral propeller flap is safe/effective in elderly; high survival, less edema, good function, high satisfaction.
Karagergou E 2025	Prospective case series	Greece	8	Mean 63.2 ± 11.0 y (43–74)	4 M/4 F	Wound breakdown and hardware exposure after ORIF of low-energy distal tibia/ankle fractures	Distal tibia diaphysis (*n* = 4) Ankle (*n* = 3) Combined distal tibia/ankle (*n* = 1)	Local perforator-based propeller flap (posterior tibial perforator, *n* = 7; peroneal perforator, *n* = 1) ± Ilizarov external fixation if non-union	Not specified numerically (local rotation)	2 years	Local propeller flaps reliably salvage coverage; 75% union at 2 y; appearance/function scores modest; consider timing/hardware strategy.
Yassin AM 2025	Retrospective multicenter cross-sectional study	Sudan (Omdurman Teaching Hospital, Atbara, Elobied, Kassala Teaching Hospitals)	31	Majority between 30–40 years (38.7%); only 1 patient >60 y (3.2%)	26 M (83.9%), 5 F (16.1%)	Weapon injuries (67.7%), road traffic accidents (19.4%), chronic ulcers (9.7%), domestic injury (3.2%)	Distal leg (15; 48.3%), foot (13; 41.9%), ankle (3; 9.7%); right side more frequent (64.5%)	Lateral supra-malleolar flap (distal-based)	Not numerically specified; pivot point ∼5 cm superomedial to lateral malleolus	6 months	The lateral supra-malleolar flap is a reliable option for distal leg and foot reconstruction. It ensures good texture and color match, preserves major vessels, and provides satisfactory functional recovery and high patient satisfaction, with manageable complication rates.
Tao S 2025	Case series	China	9 patients, 11 flaps	11–88 years (mean 46.3)	7 M, 2 F	Melanoma (*n* = 3), burns (*n* = 2), chronic neurotrophic ulcers (*n* = 3), trauma (*n* = 3)	Plantar heel (*n* = 7), plantar heel + lateral heel (*n* = 2), plantar heel + medial heel (*n* = 2)	Axial medial plantar rotation (AMPR) flap, based on medial plantar artery (MPA) or medial plantar artery perforators (MPAPs)	Rotation designed along medial plantar vascular axis; up to 180° depending on perforators; inferior margin incised partially/fully to enhance mobility	Mean 7.6 months (range 1–18)	The AMPR flap with MPAPs is reliable for small–medium plantar heel defects; the AMPR with MPA can cover larger defects but is more prone to necrosis. Advantages include easier dissection, preservation of major plantar vessels, donor-site closure without grafting, and good sensory and aesthetic outcomes.
Yavari A 2025	Case report	Iran	1	35 y	Male	Severe leg and ankle trauma from car accident with tibia and fibula fractures, hardware exposure after ORIF	Ankle joint and distal leg, 11 × 18 cm defect with exposed bone and plate	Fasciocutaneous propeller flap based on posterior tibial artery perforator	Not quantified	2 months	Propeller flaps are a valuable alternative to free flaps in lower limb reconstruction. Venous congestion, the most common complication, can be prevented with retrograde venous supercharging of the great saphenous vein, which proved safe and effective in this case.
Yang Y 2024	Retrospective clinical study	China	40 DFD patients + 43 non-DFD patients (total 83); of these, 40 DFD underwent flap reconstructions	Mean age ∼56.5 years in DFD vs. 57.8 in non-DFD	DFD: 18 male, 13 female; Non-DFD: 25 male, 18 female	Diabetic foot ulcers	Foot dorsum, ankle, plantar/sole, heel; often with toe ray amputations	Multiple flaps used: 34 ALTP (anterolateral thigh perforator, including polyfoliate and chimeric), 3 MSN (medial sural neurotrophic), 2 PTAP (posterior tibial artery perforator propeller flap), 2 MP (medial plantar), 2 Keystone	Only PTAP was a propeller flap; ALTP and others were free or pedicled flaps	24–36 months (varied by case)	Free ALTP flap effective for large, irregular DFD wounds but higher risk of necrosis vs. non-DFD; pedicled flaps (PTAP, MSN, MP, Keystone) safer in selected cases; preoperative vascular assessment crucial
Ota M 2024	Retrospective single-center comparative study	Japan	33 patients, 38 flaps (PPF: 18 in 15 patients; FF: 20 in 18 patients)	Mean 58 years in both groups (range 21–95 PPF, 27–85 FF)	PPF: 9 male, 6 female; FF: 14 male, 4 female	Trauma-related: open fractures (tibial shaft, distal tibia, ankle, calcaneus, Lisfranc, metatarsal), postoperative wound necrosis, postoperative infection	Distal third of leg, heel, mid-foot, ankle	PPF: perforator propeller flaps (mainly posterior tibial/peroneal artery perforators, mean rotation 145°); FF: ALT (13) and latissimus dorsi (7) free flaps	PPF: rotation up to 180° (true propeller flaps); FF: free transfer	Mean 31 months (PPF) vs. 24 months (FF)	Perforator-based propeller flaps (PPF) avoids microsurgery but has high venous congestion, necrosis, and delayed osteomyelitis risk. FF provides safer long-term coverage despite longer OR time. Surgeons must be cautious considering PPF as “simpler”
Katpar H 2023	Case series	Pakistan	14	Mean 33.5 ± 8.76 years	12 male (86%), 2 female (14%)	Mostly trauma (11/14, 85%)	Around knee and proximal leg	Pedicled medial sural artery perforator (MSAP) flap	Pedicled propeller-type flap, pivoting on perforator location (distal-most perforator ∼12.7 cm from popliteal crease, usually near gastrocnemius musculotendinous junction)	Weekly for first 2 weeks, then at 1, 3, and 6 months	MSAP flap is a reliable, thin, long pedicled fasciocutaneous flap with low donor-site morbidity and good aesthetic/functional results
Gatto A 2023	Case series	Italy	5 patients with Gustilo IIIB open fractures	Mean 33.8 years; all male; 1 heavy smoker	Male (100%)	Gustilo IIIB open fractures of the lower limb	Distal lower limb (tibial fractures)	Perforator-based propeller flaps for immediate reconstruction in the acute phase	Propeller rotation (angle not specified)	Mean 5.5 months	First report in the literature of immediate propeller perforator flap reconstruction for acute Gustilo IIIB lower limb fractures; found to be feasible, safe, and reliable
Ye S 2024	Retrospective study	China	34	Range 28–72 years (examples: 28, 39, 48, 70, 72 in case series table)	2 M, 9 F	High-energy trauma: 21 traffic accidents, 7 industrial injuries, 6 falls	Distal third tibia, ankle, foot soft tissue defects with exposed bone/tendon	Two types of perforator pedicle propeller flaps: • Peroneal artery perforator flap (PAPF, *n* = 18) • Posterior tibial artery perforator flap (PTAPF, *n* = 16)	Propeller-style rotation around identified perforator vessels (true island propeller flaps)	11–28 months (mean 19 months)	Both PAPF and PTAPF are safe and effective options for delayed coverage after Gustilo IIIB fractures when combined with Masquelet technique and VSD. They provide reliable flap survival, bone healing, and ankle function
Chen Y 2023	Case report	Cina	1	59 y	F	Ischemic diabetic heel ulcer con osteomielite calcaneare, risultante da ustione e complicata da grave ischemia periferica (W3 I3 fI2)	Heel ulcer with bone exposure	Peroneal artery perforator flap applied after revascularization	True propeller flap: islanded and rotated around the peroneal perforator pedicle	Up to 24 weeks	Two-stage management (revascularization + propeller peroneal artery perforator flap) is effective for ischemic heel ulcers in diabetes, ensuring durable coverage, healing, and functional recovery
Chaudhuri GR 2023	Prospective interventional observational study	India	30	16–63 years (mean 42.7)	20 male (70%), 10 female (30%)	Soft tissue defects of distal third of the leg due to trauma, infections, etc.	Perforator propeller flaps: 18 posterior tibial artery perforator flaps, 12 peroneal artery perforator flaps	Distal third of the leg, including tendo-Achilles region and medial/lateral malleoli	180° in 24 cases (80%); 90° in 6 cases (20%)	NR	Propeller perforator flaps provide reliable, versatile coverage for distal third leg defects, with minimal donor-site morbidity, shorter operative time than free flaps, and preservation of major vessels
Ran X 2022	Retrospective case series	China	10	Median 47 years (range 6–71)	8 M, 2 F	Malignant tumor (5 cases), trauma (3), postburn contracture (1), diabetic foot ulcer (1)	Pretibial (1), distal lower limb/Achilles tendon (3), dorsum of foot/lateral malleolus (4), heel (1), plantar foot (1)	Perforator propeller flap sequential transfer technique: • 1st flap: peroneal artery perforator propeller flap (14 × 4 cm to 29 × 8 cm) • 2nd flap: another perforator propeller flap to cover donor site (7 × 3 cm to 19 × 7 cm)	True propeller-style rotation around perforator vessels	2–39 months (median 15.5 months)	Sequential propeller perforator flaps are safe, effective, and reliable for distal lower limb soft tissue defects.
Noaman HH 2022	Retrospective clinical study	Egypt	85	Mean 34.7 years (range 5–70)	58 M, 27 F	Trauma (majority), infection, burn, tumor excision	Around the ankle (malleolar region, heel, anterior/posterior ankle)	Multiple: 32 free flaps, 21 distally based sural artery flaps, 12 perforator-based propeller flaps, 8 rotational local flaps, 10 skin grafts, 2 contralateral sural flaps	Propeller flaps rotated around perforator (typically 90–180°); other flaps as per type	Mean 15 months (range 6–36)	Multiple reconstructive options are available for ankle defects; propeller perforator flaps are reliable for moderate-size defects, while free flaps are best for larger or complex wounds. Each method showed good long-term functional and aesthetic outcomes with acceptable complication rates
Jin W 2022	Case series	China	8	Range 19–58 years (mean ∼36)	6 M, 2 F	High-energy trauma with large lower extremity soft-tissue defects, some with bone/tendon exposure	Distal tibia, ankle, and foot	Parallel cross-leg free flap combined with posterior tibial artery perforator pedicle propeller cable bridge flap	Posterior tibial artery perforator pedicle flap rotated as a propeller flap to create a cable bridge between limbs	6–18 months (mean ∼10)	Combining cross-leg free flap with a posterior tibial artery perforator propeller flap as a vascular cable bridge is safe, ensures flap survival, and provides effective salvage for complex lower limb wounds
Gupta S 2022	Retrospective study	India	28	Mean 29.3 ± 14.9 years	15 M (53.6%), 13 F (46.4%)	Trauma in 26 (92.9%), cellulitis in 2 (7.1%)	Lateral (39.3%), medial (35.7%), anteromedial (10.7%), anterior (7.1%), anterolateral (7.1%)	Perforator propeller flaps: 17 peroneal artery, 11 posterior tibial artery	Mean 174° ± 11.9 (range 140–180); mostly 180°	Mean 1 month (short-term)	Propeller flaps are reliable but technically demanding for distal leg reconstruction. Complication rate higher in early cases, confirming a steep learning curve; outcomes improve with surgical experience
Eldahshoury T 2021	Case series	Egypt	23	25–62 years (mean 45.5)	20 male (87%), 3 female (13%)	Mostly posttraumatic (20), postinflammatory (2), post tumor excision (1)	Lower third leg (13), leg & ankle (4), ankle (2), ankle & foot (2), heel (2)	Posterior tibial artery perforator propeller flap (11 cases); Peroneal artery perforator propeller flap (12 cases)	Classic propeller design; rotation based on selected perforator; some cases required venous supercharging	7–13 months (mean 12)	PPFs are safe, cost-effective, and reliable for distal leg/ankle defects; they preserve distal vascularity, can be performed in resource-limited settings, and serve as a viable alternative to free flaps
Tapan M 2021	Case series	Turkey	11	9–64 years	9 M, 2 F	Avulsion (2), gunshot (1), crush injury (3), diabetic foot (1), post-orthopedic surgery (2), tumor resection (1), electrical burn (1)	Heel, malleoli (medial/lateral), ankle (anterior/lateral), dorsum/plantar foot	Peroneal perforator propeller sural (PPPS) flap. Variants: direct propeller (8), interpolation propeller (2), passing-through style (1). In 2 cases, combined with posterior tibial artery perforator flap. Sural nerve coaptation in 1 case	Propeller rotation around peroneal artery perforator; extended, interpolation, and passing-through insetting styles	4–28 months	PPPS flap is versatile (direct, interpolation, passing-through, extended styles), can be combined with posterior tibial flap, provides reliable coverage and sensory potential, with low complication rates
Wang P 2021	Retrospective single-center study	China	82	Mean 36.5 years (range 18–65)	47 M, 35 F	Trauma (62 cases, 75.6%), infection (20 cases, 24.4%)	Medial malleolus (31), external malleolus (8), lower tibia (32), middle tibia (11)	Propeller perforator flaps based on posterior tibial artery (62), anterior tibial artery (12), peroneal artery (8)	150–180° in most cases; necrosis rate significantly higher when rotation >150°	Mean 12.5 months (range 3–36)	Effective safe distance between perforator and wound edge is ≥3.5 cm. When <3.5 cm, necrosis risk increases markedly (AUC 76.1%, sensitivity 69.7%, specificity 82.4%). Rotation >150° also associated with higher necrosis risk
Mallett KE 2021	Retrospective cohort study	USA	44	Mean 53 ± 18 years	31 F, 13 M	Resection of soft tissue sarcomas (various histologies: myxofibrosarcoma 10, leiomyosarcoma 8, UPS 5, synovial sarcoma 5, clear cell sarcoma 3, others 13)	Foot and ankle subunits (toes, plantar forefoot, midfoot, heel, malleoli)	21 free flaps (gracilis, latissimus, ALT, serratus, VRAM, radial forearm); 13 pedicled flaps (soleus, sural artery, posterior tibial, medial plantar, abductor digiti minimi); 10 rotational perforator (propeller) flaps	Rotational perforator flaps, random pattern (propeller-type)	Mean 10 ± 7 years	Flap reconstruction (including propeller flaps) is essential for limb salvage in foot/ankle STS. High limb salvage (84% at 10 years), acceptable function, and comparable outcomes between free and local/propeller flaps
Shih YJ 2023	Retrospective case series	Taiwan	15	Mean 53.7 years (range 22–89)	10 M, 5 F	Diabetic foot (6), chronic osteomyelitis (2), infected bursitis (1), trauma (6)	Lateral malleolus region (ankle)	Peroneal artery perforator flap. Two categories: • Propeller flap (8 cases, rotated up to 180°) • Rotation flap (7 cases, supplied by perforator + random base circulation)	Propeller group: true perforator-based pivot rotation up to 180°	Mean 30 months (range 3–60)	Peroneal artery perforator flaps (propeller and rotation types) are reliable for small-to-moderate lateral malleolus defects. Propeller-type allows greater mobility, while both show low morbidity and good long-term results
Guillier D 2021	Retrospective study	Switzerland	21	Range 20–87 years (examples: 20, 29, 45, 80)	NR	Open/closed fractures with fixation, chronic infection, septic arthritis, osteomyelitis	Distal third of the leg, ankle, calcaneus, patella, tibia	Propeller perforator flaps (PPFs) based on peroneal, posterior tibial, or lateral genicular arteries	True propeller flap, rotated 90–180° on selected perforator	Mean 15 ± 7 months (F group) vs. 20 ± 13 months (NF group)	PPFs are effective for small-to-medium defects, but when hardware is present, risk of complications and major revisions is higher. Free flaps may be safer in those cases
Yadav P 2021	Prospective interventional single-center study	India	53	14–65 years (mean 32.7)	47 M, 6 F	Trauma (41), electric burn (8), pressure ulcers (2), post-cellulitis (1), squamous carcinoma (1)	Lateral malleolus (15), anterior ankle (10), medial malleolus (8), Achilles tendon region (6), combined defects (14)	Perforator-based propeller flaps: peroneal artery (35), posterior tibial artery (18)	90–180°	Minimum 6 months	Propeller flaps are safe and reliable for small-to-medium distal leg, ankle, malleolar, and Achilles defects. Low donor morbidity, good stability; minor necrosis manageable with skin graft
Kim K 2021	Retrospective cohort study	Republic of Korea	35	Mean 61 years (range 30–79)	24 M, 11 F	Diabetic foot ulcer (20), trauma (4), bursitis (2), burn (2), cellulitis (2), oncologic resection (2), venous ulcer (1), chronic ulcer (1), postop orthopedic complication (1)	Lateral malleolus (15), heel (10), anterior tibia (6), medial malleolus (2), foot dorsum (2)	Propeller perforator flaps: peroneal artery (22), posterior tibial artery (10), anterior tibial artery (3)	150–180°	Minimum 1 month; mean follow-up not specified	Diabetic patients had significantly higher complication rates (∼60%) vs. nondiabetic (∼13%). Risk factors: female sex, diabetes, chronic renal failure, and diabetic neuropathy. Authors conclude propeller perforator flap may not be effective for diabetic foot ulcers
Zang M 2020	Retrospective case series	China	9	Mean 36.4 years (range 6–61)	7 M, 2 F	Trauma, tumor excision (melanoma, sarcoma, carcinoma), chronic ulcer, diabetic foot, prosthesis exposure	Pretibial (1), distal leg/Achilles tendon (3), dorsum foot/lateral malleolus (3), plantar foot (1), heel (1)	Peroneal artery perforator propeller (PAPP) flap for primary defect + relay propeller flap (from peroneal, medial sural, or lateral sural perforators) for donor site closure	Primary PAPP flaps: 90–180°; Relay flaps: 120–180°	Mean 23 months (range 13–39)	Propeller flap relay technique enables harvesting of large PAPP flaps without major donor-site morbidity; both defect and donor site covered with local propeller flaps in one stage
Demiri E 2020	Retrospective comparative study	Greece	54	Mean: 59.1 y (NCF) vs. 50.8 y (PF)	44 M, 10 F	Trauma (21), chronic ulcers (26), previous surgery (7)	Achilles zone (16), posterior heel (14), dorsum of foot (9), malleoli (8), anterior ankle (5), lateral foot (2)	Group A: Reverse neurocutaneous flaps (19 sural, 15 lateral supramalleolar); Group B: Propeller perforator flaps (13 peroneal, 7 posterior tibial)	Propeller flaps rotated up to 180°	6–59 months	Both techniques are safe for diabetic foot reconstruction. NCF better for large/distal defects but with longer healing and higher revision needs; PF better in younger patients, proximal/smaller wounds, with faster mean healing (40.7 vs. 48.1 days)
Zhao W 2020	Retrospective clinical series	China	31	Mean 36 years (range 12–53)	21 M, 10 F	17 acute traumatic heel wounds; 14 chronic infectious wounds or ulcers	Heel region	Peroneal artery perforator propeller flap, with digital 3D flap planning using CTA and Mimics19.0 software	Classic propeller design (rotated around perforator pivot)	3–18 months (average 12 months)	Digital planning via 3D modeling improves flap design accuracy, reduces surgical difficulty, and lowers risk from vascular anatomical variations
Franchi A 2020	Retrospective case series	Switzerland	8	Mean 54 years (range 34–76)	4 M, 4 F	Post-traumatic soft tissue defects (acute = 5, chronic = 3)	Distal leg (4), heel (2), proximal lateral thigh (1), proximal posterior thigh (1)	Sequential perforator-based propeller flaps: first flap for primary defect, second flap for donor site closure	Each flap rotated 90–180° around skeletonized perforator	Median 7 months (range 2–12)	Sequential propeller flaps allow complete, like-with-like wound closure, avoiding skin grafts or free flaps in selected cases. Technique requires careful planning, ICG use, and high surgical expertise
Kosutic D 2020	Retrospective case series	United Kingdom	17	Mean 57 years (range 29–82)	7 M, 10 F	Reconstruction after wide local excision of melanoma around the knee	Knee/peripatellar region	Distal perforator-only propeller ALT flap (D-POP), based on the most distal ALT perforator (“D”)	Propeller rotation; perforators dissected intramuscularly or intraseptally to allow mobilization	4 years	Technique is simple, reliable, and versatile. D-POP ALT flaps are thin, pliable, allow primary donor-site closure, and represent a superior alternative to gastrocnemius muscle flaps, avoiding muscle sacrifice and morbidity
Wang W 2020	Retrospective clinical series	China	18	Average 32.8 years (range 8–56)	12 M, 6 F	Traffic accidents (11), falls from height (3), heavy object injuries (4)	Dorsum of foot (9), heel (4), lateral malleolus (5)	• Recipient defect covered by peroneal artery terminal perforator propeller flap (size ∼6 × 3 to 18 × 7 cm) • Donor site closed using anterior tibial artery perforator propeller flap (size ∼8 × 3 to 16 × 6 cm)	Both the peroneal artery terminal and anterior tibial artery perforator flaps were islanded on a single perforator and rotated 90–180° depending on defect site.	6–15 months (mean ∼12.5 months)	This relay technique provided stable coverage of foot and ankle defects with good venous patency, minimal complications, excellent sensory recovery, and high functional scores (AOFAS 100% excellent/good).
Innocenti M 2019	Retrospective comparative study	Italy	179	Free flaps: mean 49 years (range 5–89). Propeller flaps: mean 53 years (range 11–92)	Free flaps: 61 male/39 female. Propeller flaps: 46 male/33 female	Post-traumatic (49% free; 50.6% propeller), oncologic resections (28% free; 26.6% propeller), pressure sores (17% free; 17.7% propeller), infectious (3% free; 5.1% propeller), burns (3% free; none propeller)	Lower limb: leg, ankle, foot (not further specified per group)	Free perforator flaps: mostly ALT (81%), SCIP (7%), radial (7%), medial plantar (4%), ulnar (1%). Propeller flaps: posterior tibial perforator (46%), peroneal (14%), popliteal (10%), deep femoral (10%), anterior tibial (6%), medial plantar (5%), circumflex femoral (5%), saphenous (4%)	Propeller flaps rotated on perforators, typically 90–180° (not quantified in paper)	Mean 12 months	Both techniques are effective. Free flaps remain standard for large or complex defects; propeller flaps are best for small/medium, otherwise healthy extremities. Propeller flaps showed slightly higher complication rates but lower failure rate. Cost analysis strongly favored propeller flaps (€1595 vs. €5077 per patient), with shorter operative time (117 vs. 354 min) and hospitalization (4 vs. 8 days)
Yu D 2019	Clinical case series	China	13	Mean ∼40 years (range 14–65)	9 M, 4 F	Traffic accidents (7), crushing injuries (4), falling from height (2)	Soft tissue defects of the knee	Free-style perforator flaps: 9 propeller flaps, 6 rotating flaps, and 2 V-Y advancement flaps; some cases used combined flaps	They used “propeller” flaps among the flap types, which implies rotation around the perforator — though exact degree of rotation isn’t specified in the abstract.	3–24 months, average ∼6 months	Free-style perforator flaps permit maximal use of nearby donor tissue with reliable blood supply, minimal trauma, simple operation, good healing, and satisfactory functional and aesthetic results.
Shahabuddin SF 2020	Retrospective single-center clinical study	India	40	Mean 25.4 years (range 8–60)	32 M, 8 F	Trauma 60%, implant-exposed wounds 15%, post-infective non-healing ulcer 10%, post-traumatic unstable scar 5%, post-repair exposed tendoachilles 5%, electrical injury 5%	Lower third leg 50%, middle third leg 30%, upper third leg 10%, heel pad/tendoachilles 10%	Perforator-based propeller flaps (posterior tibial artery perforator 65%, anterior tibial 15%, peroneal 10%, medial sural 10%)	Group I: 150–180° (20 patients). Group II: 90–150° (20 patients)	Mean 12 months	Perforator-based propeller flaps are safe, reliable, and effective for leg reconstruction, especially distal third. Risk of necrosis higher at 180° rotation, improved outcomes with meticulous perforator dissection. Suitable as first-line option when defect parameters allow
Dhar LK 2019	Prospective observational study	Bangladesh	9	16–65 y	9 M, 3 F	Mostly post-traumatic wounds (≈ 66.7%), plus infective, malignancy excision, electric burn wounds	Defect sizes ranged from 2 × 2 cm up to 7 × 5 cm; exposed bone or tendon in lower leg and ankle region	Posterior tibial artery perforator-based propeller flap; local flap, no microvascular anastomosis	Between 145–180°, mean ≈ 163°	Up to 6 weeks	Posterior tibial artery perforator flaps (including propeller type) are reliable for small-to-medium lower leg and ankle defects. Careful design, perforator dissection, and venous drainage management are essential to avoid necrosis/congestion. Cost-effective alternative to free flaps in resource-limited centers
Kelahmetoglu O 2018	Case report	Turkey	1	35 y	F	History of tibial and fibular fractures with fixation after traffic accident; wound dehiscence over Achilles tendon following free latissimus dorsi musculocutaneous (LDMC) flap	Achilles tendon region	Propeller perforator flap harvested from a previously transferred LDMC free flap (“backup flap”)	150° clockwise rotation	12 months	Multidetector CT (MDCT) enabled precise identification of perforators within a previously transferred musculocutaneous flap, allowing safe planning of a propeller perforator flap without creating a new donor site, and achieving reliable coverage in a complex traumatic region
Song D 2017	Prospective clinical series	China	12	Range 14–52 years, mean 23.4 years	10 M, 2 F	Motorcycle spoke injuries (7 cases), traffic accidents (5 cases)	Foot and ankle soft tissue defects	Primary defect: distally based sural flap. Donor site of sural flap reconstructed with relaying lateral gastrocnemius artery perforator propeller flap	90–180°, mean 110.45°	6–14 months, mean 12.4 months	Combining a distally based sural flap with a relaying lateral gastrocnemius artery perforator propeller flap avoids skin grafting at the donor site, reduces morbidity, preserves calf function, and provides reliable and aesthetic reconstruction for foot and ankle defects
Shen L 2017	Retrospective observational clinical study	China	36	Mean 39.7 years (range 6–83)	29 M, 7 F	All defects due to trauma	Heel (20 cases, 55.6%), ankle (7), distal lower leg (5), dorsal foot (4)	Peroneal perforator pedicle propeller flap; perforator identified preoperatively by duplex Doppler; flap islanded and rotated around perforator	Up to 180°; mean pivot point distance ∼10.1 cm from lateral malleolus	Not explicitly long-term; flaps checked until wound healing (all eventually cured)	Peroneal perforator propeller flaps are a reliable option for distal leg, ankle, and heel defects, with good cosmetic and functional outcomes. Venous congestion is the main complication, but can be managed by delayed closure or bleeding therapy. Preoperative Doppler mapping is crucial for safety
Chaput B 2018	Prospective comparative clinical study	France	60 patients: 30 conventional propeller perforator flaps (PPF) vs. 30 venous-supercharged PPF (vsPPF)	PPF: mean 51 years (range 15–72). vsPPF: mean 54 years (range 21–85)	NR	Mainly acute trauma (≈67% PPF, 60% vsPPF), also chronic osteitis, oncologic resections, burns	Mostly middle and distal third of leg (>80%), ankle, Achilles tendon	Posterior tibial artery perforator (PTAP) or fibular artery perforator (FAP) based propeller perforator flaps; vsPPF included distal venous anastomosis (supercharging)	Mean: PPF 126.7° ± 33.1; vsPPF 121.3° ± 31.9 (not significantly different)	NR	Venous supercharging significantly reduces venous congestion, distal necrosis, and complication rate in lower limb propeller perforator flaps. vsPPF increased operative time but decreased hospital stay and improved flap reliability
Özalp B 2016	Retrospective observational case series	Turkey	7	2–13 y (mean 6.7 y)	7 M	Burn injuries (*n* = 4), traffic accidents (*n* = 3)	Peri-ankle (4), knee (1), anterior lower tibia (1), foot dorsum (1)	Perforator-based propeller flaps: posterior tibial artery (*n* = 4), anterior tibial artery (*n* = 2), descending branch of lateral circumflex femoral artery (*n* = 1)	90–180°	5–36 months (mean 13 months)	Propeller perforator flaps are safe and reliable for leg reconstruction in children, with complete survival and high satisfaction. Meticulous dissection required due to small vessel diameter. Pediatric age is not a risk factor for flap survival
Cadenelli P 2015	Case report	Italy	1	52 y	100% M	Wide resection of soft-tissue sarcoma of the anterior-lateral knee after radiotherapy	Knee, anterior-lateral aspect	Proximally based ALT flap, used with advancement and propeller rotation (propeller perforator flap, type C in Yu’s system)	180° rotation around the distal perforator	6 months	In cases of knee soft-tissue defects with compromised vascular web, a proximally based ALT flap with propeller advancement offers safe, thin, pliable coverage, preserving vascular trunks and avoiding free flap morbidity
Kang JS 2016	Case report	South Korea	1	45 y	100% M	Traffic accident → popliteal artery occlusion, tibial fracture, compartment syndrome → fasciotomy; subsequent heel pressure sore	Heel, with fasciotomy scar on lower leg	Pedicled propeller perforator flap: attempted peroneal artery (PA) perforator, but inadequate → posterior tibial artery (PTA) perforator used; flap rotated to cover heel	180° clockwise rotation	3 months	Even in the context of major vessel injury and fasciotomy, a propeller perforator flap can provide reliable heel reconstruction if distal flow is intact. Propeller flaps are valid alternatives to free flaps when microsurgery is high-risk
Ademola SA 2015	Case report	Nigeria	2	Both 34 years	100% M	Gunshot injuries (Gustilo IIIB open fractures)	Distal third of the leg (case 1: medial and lateral defects; case 2: posterior wound with exposed Achilles tendon)	Perforator-based propeller flap (from the tibioperoneal system, medial and lateral)	Flaps rotated 180° like propeller blades	Around 6 weeks until clinical healing (no long-term follow-up reported)	Propeller perforator flap is a simple, fast, reliable, and cost-effective option for distal leg reconstruction, especially when the reverse sural flap is not feasible
Horta R 2014	Case report	Portugal	1	50 y	M	Open tibial fracture (middle third) after tractor accident; complicated by infection and bone exposure	Right leg, middle third (tibia)	Perforator-based propeller flap (fasciocutaneous) based on two posterior tibial artery perforators; venous supercharging with tributary of great saphenous vein	90° rotation	2 months	A propeller perforator flap with venous supercharging is a safe and effective option for leg reconstruction with exposed bone, improving reliability by preventing venous congestion
Cinpolat A 2014	Retrospective case series	Turkey	6 patients, 7 flaps	Mean 37.6 years (range 13–55)	5 M, 1 F	Electrical injury (4 patients), benign tumor (2 patients)	Distal foot: first metatarsophalangeal joint (3 cases), 5th MPJ (1), 5th metatarsal head (1), dorsum of 4th toe (2)	Distal foot: first metatarsophalangeal joint (3 cases), 5th MPJ (1), 5th metatarsal head (1), dorsum of 4th toe (2)	45–180° depending on perforator location	Mean 4.2 months (range 1–6)	MAP-based propeller flaps are safe and reliable for small-to-moderate distal foot defects with exposed tendon/bone; provide thin, pliable coverage without sacrificing major arteries
Dong KX 2014	Retrospective clinical series	China	20	Mean 28 years (range 5–75)	12 M, 8 F	5 spoke injuries, 4 postoperative infection with necrosis, 2 heavy object trauma, 9 car accidents	Lower leg and foot (soft tissue defects 2 × 8 to 10 × 20 cm)	Perforator pedicled propeller flaps: 15 peroneal artery perforator flaps, 5 posterior tibial artery perforator flaps	≤180° (rotation chosen to minimize pedicle stress; clockwise or counterclockwise)	5 × 11 cm to 12 × 28 cm	Perforator pedicled propeller flaps are simple, safe, reliable for lower leg/foot defects. Preoperative Doppler localization critical. Complication rate low; main risk is venous crisis when pedicle rotated near 180°
Innocenti M 2014	Case series	Italy	5	26–72 years	100% M	3 trauma, 1 septic complication after elective knee surgery, 1 infection after prosthesis	Knee, involving proximal tibial metaphysis, patella, distal femur	Chimeric pedicled flap: medial gastrocnemius muscle flap + fasciocutaneous propeller flap based on medial sural artery perforator	90–180° depending on defect location; propeller skin paddle oriented independently from muscle	Mean 3–6 months; case example shown at 3 months	Combining medial gastrocnemius with a propeller fasciocutaneous paddle increases coverage surface and flexibility, enabling coverage of complex knee defects without resorting to free flaps. Best indicated for large anterior knee defects involving both tibial tuberosity and patella
Chang SM 2014	Retrospective clinical study	China	24	3 Age Mean 30 years (range 20–60)	100% M	Trauma (19), post-reconstruction defects (5)	Foot and ankle defects, especially heel and dorsum of foot	Distally based **perforator propeller sural flap**	160–180° (propeller type)	6–12 months	Propeller perforator flap offers reliable reconstruction for complex foot and ankle defects, with excellent survival and functional outcomes. Venous congestion can be managed with appropriate flap design.
Tos P 2011	Retrospective case series	China	28	19–67 years	23 M, 5 F	Trauma (38%), chronic ulcers (28%), post-surgical complications (21%)	Lower leg (distal third) and foot (dorsum, heel, ankle)	Perforator-based propeller flaps (posterior tibial perforator, peroneal perforator)	90–180° (depending on perforator location)	Follow-up 6–12 months (mean ∼9 months)	Perforator-based propeller flaps are reliable for soft tissue defects in the lower limb, with good flap survival and low complication rates when applied to small/medium defects
Rubino C 2009	Case report	Italy	1	48 Y	M	Chronic osteomyelitis of the right fibula	Right distal fibula (lower leg)	Local propeller perforator flap based on a perforator of the peroneal artery	Approximately 120° rotation to cover the defect	12 months	The propeller perforator flap is a reliable option for covering small-to-moderate defects in chronic osteomyelitis of the lower extremities, reducing donor site morbidity and providing stable soft tissue coverage
Pignatti M 2008	Prospective case series	Italy	6	15–63 years (mean 52.5 years)	5 M, 1 F	Post-traumatic tibial fractures (4), post-traumatic malleolar fracture with Achilles tendon disruption (1), prosthesis infection with prior failed coverage (1)	Leg (tibial bone exposure), knee joint, Achilles tendon, medial/lateral malleolus	Propeller perforator-based flap, skeletonized perforator flap, skin island with 2 blades (size 8 × 9 cm to 25 × 12 cm)	90–180° around the perforator; dependent on defect location	6 months	Skeletonized propeller perforator flaps provide reliable coverage of complex leg and knee defects, allow partial donor-site coverage with the flap itself, maintain good perfusion, and minimize donor-site morbidity. Flap design must consider prior scars and perforator location to optimize the propeller blades.
Moscatiello F 2007	Case series	Spain	6	Mean age was 55.5 years	5 M, 1 F	4 post-traumatic cases and 2 oncologic resections, located in the peripatellar region and upper thigh.	Peripatellar region, anterior/upper leg	Propeller distal anteromedial thigh perforator flap, single perforator-based, rotated to cover defect	180° around the single perforator	1–4 years	The distal anteromedial thigh is a reliable donor site for propeller perforator flaps. A single adequate perforator is sufficient, minimizing donor-site morbidity. The flap provides a versatile option for reconstructing knee and upper leg soft tissue defects.

**Table 3:** Descriptive of selected studies: functional, clinical, and patient-reported outcomes of propeller perforator flaps for lower-extremity reconstruction.

First author, Year	Flap survival	Venous patency	Flap swelling	Functional clinical/radiological outcomes	Complications	PROM (patient-reported outcome measure)
Chen G 2025	14/15 complete survival (93.3%); 1 distal superficial necrosis healed conservatively	100% patency on Doppler at 1 month	At 7 days, flap swelling was observed in 6 grade I and 9 grade II cases, while at 6 months it decreased to 13 grade I and 2 grade II cases.	AOFAS ankle–hindfoot at 6 mo: 8 excellent, 5 good, 2 fair (86.7% excellent/good)	Only 1 minor superficial distal necrosis; no reoperations	Appearance satisfaction: all satisfied at 6 months
Karagergou E 2025	All flaps survived; 1 transient venous congestion treated with leeches	Not reported (clinical viability maintained)	NR	Bony union 6/8 (75%) at 2 y	1 venous congestion (resolved); 1 donor-site delayed healing	LIMB-Q™ at 2 y: Appearance 61 ± 21; Function 73 ± 22; Expectations 74 ± 15; others >80
Yassin AM 2025	30/31 successful (96.7%)	Not assessed instrumentally; clinical flap viability reported	1 case (3.2%) of flap edema	Most patients without fractures regained independent walking and shoe-wearing at 4 months; those with fractures required delayed mobilization until bony healing	6 partial necrosis (19.4%), 1 complete necrosis (3.2%), 4 infections (12.9%), 1 donor skin graft loss (3.2%), 1 flap edema (3.2%)	Aesthetic satisfaction: 28/31 (90.3%) rated good, 3/31 (9.7%) rated poor (all female)
Tao S 2025	9/9 MPAP flaps survived; 2/2 MPA flaps had complications (1 marginal necrosis, 1 partial necrosis, both healed with secondary procedures) → overall survival 100%	Not directly assessed, clinical viability confirmed	Not systematically reported	Sensation preserved in arch, reduced in medial plantar heel (partial recovery over time); all flaps provided stable plantar coverage suitable for ambulation; 1 recurrent ulcer at 12 months resurfaced successfully with second flap	1 marginal necrosis (managed with sutures), 1 partial necrosis (skin graft), 1 recurrent ulceration (successfully re-covered)	NR
Yavari A 2025	Complete survival, no necrosis	Normal color and capillary refill maintained; retrograde venous anastomosis effective in preventing congestion	NR	Defect healed completely at 2 months; stable coverage, no mention of gait or functional score	NR	NR
Yang Y 2024	ALTP group: 2 total necrosis, 4 partial necrosis; other flap groups: no necrosis	Venous congestion noted in 2 ALTP cases	NR	At 12 months: 12 excellent, 16 good, 8 fair, 3 poor walking ability among DFD ALTP cases	Higher in DFD vs. non-DFD: total necrosis (2), partial necrosis (4), infections/hematoma; none in MSN, MP, Keystone, PTAP groups	Aesthetic evaluation: non-DFD significantly better than DFD; VAS appearance not directly reported, but donor/recipient area evaluated
Ota M 2024	Success: PPF 78% (22% coverage failure), FF 95% (5% failure). Complete necrosis: PPF 11%, FF 5%. Partial necrosis: PPF 39%, FF 10%	Venous congestion: PPF 72% vs. FF 10% (significantly higher in PPF)	Reported as venous congestion/edema; managed with decompression or leeches	Both groups achieved walking ability; but PPF had higher risk of delayed osteomyelitis (20%) requiring vascularized bone grafts	PPF: higher early complications (necrosis, congestion), 4 delayed osteomyelitis; FF: fewer complications but longer operation time	NR
Katpar H 2023	All survived; only 2 patients (14%) had complications	NR	NR	Reliable coverage, good functional/aesthetic outcome	2 cases (14%), not detailed but minor	NR
Gatto A 2023	All flaps survived; no surgical revisions required; 1 superficial necrosis in the heavy smoker	NR	NR	No cases of nonunion or osteomyelitis at follow-up	1 superficial necrosis (smoker)	NR
Ye S 2024	100% flap survival. Minor complications: 2 epidermal necrosis (PTAPF), 1 superficial infection (PAPF); all healed conservatively	NR	NR	Bone union: • PAPF – mean 13.1 months, RUST 12.68, AOFAS 84.1 • PTAPF – mean 12.6 months, RUST 13.7, AOFAS 82.8	Minimal: superficial infection (1), epidermal necrosis at flap tip (2)	NR
Chen Y 2023	Successful; complete healing of the ulcer by week 7; no flap loss	NR	NR	Stable, plantigrade foot; moderate load at 12 weeks; full weight-bearing by 24 weeks	NR	NR
Chaudhuri GR 2023	26/30 successful. 2 partial necrosis (<15%), 1 complete necrosis, 1 infection	NR	NR	Stable coverage achieved; avoided free flap; preserved major vessels and muscles	13.3% overall: 2 partial necrosis, 1 complete necrosis, 1 infection	NR
Ran X 2022	All surgeries succeeded; 1st flap: 8 survived fully, 2 had venous congestion (1 partial necrosis required skin graft; 1 resolved in 7 days); 2nd flap: all survived; no hematoma or infection; primary closure achieved	2 cases of venous congestion (1 resolved, 1 partial necrosis).	NR	Good color/texture match, restored contour, no recurrence, stable function.	Minor–venous congestion, 1 partial necrosis; no hematoma/infection	NR
Noaman HH 2022	High survival; most flaps successful, few partial necrosis; overall success rate ∼95%	NR	NR	Stable coverage, preserved ankle motion, protective sensation in some flaps (especially neurocutaneous); patients could walk normally after healing	Partial necrosis in some propeller and sural flaps, infection in few cases, minor wound dehiscence; no major donor site morbidity	NR
Jin W 2022	All flaps survived; no total flap loss	Venous crisis not reported; vascular bridge with propeller flap maintained flow	NR	Stable wound coverage, satisfactory limb salvage, ability to ambulate restored in all cases	Minor: 1 partial necrosis at flap edge, healed after dressing change; no major infections	NR
Gupta S 2022	25/28 successful (89.3%); 3 total necrosis (10.7%), 2 partial necrosis (7.1%)	Venous congestion in 2 cases (7.1%), resolved with nitroglycerin patch	NR	Early stable coverage; propeller flaps provided good functional and aesthetic results	28.6% overall: 3 total necrosis, 2 partial necrosis, 2 venous congestion, 1 infection, 1 wound dehiscence	NR
Eldahshoury T 2021	21/23 successful (91.3%); 2 flap failures (1 total loss from congestion, 1 partial necrosis)	Duplex ultrasound of distal artery in 15 patients showed no significant change in peak arterial velocity post-op (49.2 → 48.4 cm/s; *p* = 0.183)	NR	Stable soft tissue coverage, no ischemic compromise, all patients achieved satisfactory wound healing	2 major (1 total loss, 1 partial necrosis needing graft); 2 minor (delayed wound healing, partial graft loss at donor site)	NR
Tapan M 2021	9/11 complete survival; 2 complications (1 major with flap loss needing free latissimus + eventual amputation; 1 partial necrosis requiring scar revision)	Venous congestion caused partial necrosis in 1 extended flap	NR	Most patients could wear shoes/boots easily post-op; sural nerve coaptation gave good sensory recovery in heel weight-bearing area	1 severe flap loss (amputation at 4 months due to osteomyelitis); 1 partial necrosis with scar revision; otherwise minimal	functional outcomes (shoe-wearing, sensation) used as surrogate
Wang P 2021	65/82 (79.3%) complete survival; 17 necrosis (11 partial, 6 total = 20.7%)	Venous crisis in 19 cases (23.2%); 4 salvaged, 15 progressed to necrosis	NR	91.5% of wounds repaired successfully with flap or secondary measures; good contour at long-term follow-up	Flap necrosis 20.7% (partial 13.4%, total 7.3%); venous crisis 23.2%; arterial crisis 2.4%; infection 13.4%	Aesthetic outcome scored: 2 excellent, 35 good, 37 fair, 5 poor, 3 very poor
Mallett KE 2021	42/44 (95%); 2 total flap losses (1 reverse sural, 1 rotational perforator), both salvaged with new flaps	NR	NR	98% ambulatory; mean MSTS-93 score 80 ± 18%; no difference between free (84%) and pedicled/propeller flaps (76%, *p* = 0.11)	43% overall (19/44): infections (9), flap necrosis (7), partial flap loss (5), total flap loss (2)	NR
Shih YJ 2023	All flaps survived; 3 cases of partial necrosis healed with skin grafting	Partial necrosis linked to venous congestion in 3 diabetic/PAOD patients	NR	NR	3 partial necroses (20%), all in elderly/diabetic/PAOD patients	NR
Guillier D 2021	Failures higher with hardware: 3 major secondary procedures in F group vs. 1 in NF group	Venous congestion was a common cause of partial necrosis and hardware exposure	NR	NR	F group: 3 major failures (including total flap necrosis, distal necrosis with hardware exposure) + 2 minor; NF group: 1 major, 3 minor (distal tip necrosis, dehiscence)	NR
Yadav P 2021	43/53 (81%) healed without complication; no total flap loss	Venous congestion in 3 patients (managed with elevation, loosening sutures)	NR	At 6 months, 40 patients reviewed: all flaps stable, no sinus or ulceration	10/53 (18.8%): distal necrosis (5), epidermolysis (2), venous congestion (3)	NR
Kim K 2021	21 complete healing (60%); 14 complications (40%)	Venous congestion in 9 patients (some leading to necrosis)	Edema and congestion described in necrosis cases	NR	14/35 patients (40%): 8 major (partial or total necrosis), 6 minor (healed conservatively)	NR
Zang M 2020	All PAPP flaps survived; 1 had venous congestion → partial necrosis needing skin graft	One venous congestion episode (resolved after partial necrosis management)	NR	Stable coverage, preserved lower leg contour, linear scars, donor-site closure without graft	1 partial necrosis in primary PAPP flap; none in relay flaps	NR
Demiri E 2020	Uneventful healing: 20/34 NCF (58.8%) vs. 12/20 PF (60%); 1 complete flap loss in each group	Distal flap necroses due to venous insufficiency: 10 NCF vs. 7 PF	Venous congestion/necrosis described, not systematically measured	All patients except one (NCF → amputation) regained ambulation	NCF: 14/34 (41.2%); PF: 8/20 (40%); revision surgeries: NCF 44.1%, PF 40%	NR
Zhao W 2020	All flaps survived; 4 venous crises and 3 partial necroses occurred but were treated successfully	4 cases of venous crisis were reported; all were managed successfully, maintaining final flap survival.	NR	AOFAS scores: 17 excellent, 11 good, 3 fair — excellent/good rate = 87.5%	3 cases of partial flap necrosis and 4 venous crises; all managed with conservative measures or minor revision, no total flap losses.	Patients regained stable heel coverage with restored walking ability.
Franchi A 2020	7/8 patients successful complete closure; 1 patient (15% necrosis of first flap) required free gracilis flap	Intraoperative ICG angiography used to confirm perfusion; one case predicted necrosis	NR	All but one patient returned to walking in normal shoes; good color match and thickness; overall excellent functional/aesthetic results	1 partial flap necrosis (≈20% of flap, converted to free flap); 1 superficial infection with hematoma managed conservatively	NR
Kosutic D 2020	Uneventful healing in all cases; no flap loss	Preserved with anterograde flow; no venous congestion problems reported	NR	All patients ambulatory immediately post-op; discharged on postoperative day 1; excellent functional and aesthetic results at long-term follow-up	None significant; no flap necrosis	Aesthetic outcome judged excellent by senior author
Wang W 2020	All recipient and donor sites healed by primary intention except one case of hemorrhagic swelling in the peroneal-based flap (resolved with treatment)	Venous patency Flap swelling Functional clinical/radiological outcomes	Only one case of hemorrhagic swelling occurred in the peroneal artery flap; it resolved with treatment. No generalized edema or persistent swelling was reported.	• Donor and recipient flaps matched well in color, thickness, and texture. • Only linear scars at donor sites. • Two-point discrimination in peroneal flaps was 10–12 mm (mean ∼11 mm). • AOFAS scores: 15 excellent, 3 good → 100% “excellent/good” rate.	One patient had hemorrhagic swelling in the peroneal artery flap, managed conservatively; no flap loss or major complication reported	Not directly reported with standardized questionnaires, but functional recovery and high patient satisfaction inferred from AOFAS scores and follow-up description.
Innocenti M 2019	Free flaps: 94% (6 total necrosis, 2 amputations). Propeller flaps: 96.3% (3 total necrosis, salvaged with free flap)	Venous congestion reported in 8% of free flaps and 5% of propeller flaps	NR	Not systematically reported; both methods achieved stable coverage and ambulation	Free flaps: 14% overall (wound dehiscence with venous stasis 8%, ischemia 5%, partial necrosis 1%). Propeller flaps: 21.5% overall (distal necrosis 16.5%, venous stasis 5%). Secondary surgery: 6% free vs. 12.7% propeller	NR
Yu D 2019	All flaps survived; incisions healed by first intention in 12/13 cases; 1 case of congestion and delayed healing	Implicitly good except for that one case with congestion; no reports of total venous failure	NR	Shape and motion of knee were satisfactory in follow-up	All 13 flaps survived. 1 patient developed venous congestion with delayed healing. The remaining 12 patients healed primarily without issues. No total flap necrosis, infection, or donor-site morbidity was reported.	NR
Shahabuddin SF 2020	38/40 successful (95%). 2 total necrosis (both in Group I, early cases)	5 patients developed venous congestion, resolved with elevation	Transient edema in 1 case	Good healing in most cases, stable coverage, acceptable cosmetic results. Patients reported complete satisfaction	Overall 10% (4/40). 2 total flap losses, 2 partial/marginal necrosis with infection or dehiscence	High satisfaction reported qualitatively
Dhar LK 2019	8 out of 9 flaps completely survived; 1 case developed marginal necrosis which healed secondarily	2 complications: transient venous congestion; superficial epidermonecrolysis (both resolved spontaneously)	Post-op edema minimized with elevation and immobilization; 1 superficial epidermolysis noted	Good skin color/texture match, patients walked normally, plantar flexion preserved	2 partial flap necrosis (16%), 1 epidermonecrolysis with congestion (8%)	NR
Kelahmetoglu O 2018	Complete survival, uneventful healing	Not reported as problematic; perfusion confirmed with preoperative MDCT imaging	NR	Stable Achilles coverage, complete healing, no recurrence of wound dehiscence	None significant; no flap necrosis	NR
Song D 2017	All flaps survived uneventfully; both recipient and donor sites healed primarily	No vascular crisis reported	No evident swelling reported	Donor sites left only linear scars; calf function not impaired; good color and contour match; patients satisfied with aesthetics and function	None significant: no vascular crisis, wound dehiscence, or major complications	Not formally assessed, but patients were satisfied with color, contour, and function
Shen L 2017	All wounds eventually healed; 2 cases of distal necrosis requiring debridement + grafting	9 patients (25%) had venous congestion; managed with delayed coverage (3 cases), bleeding therapy (5 cases), conservative observation (1 case). 7 flaps survived, 2 developed distal necrosis	Flap edema/congestion described; swelling reduced with delayed closure or bleeding therapy	Flaps had good cosmetic appearance, matched texture, allowed patients to wear socks/shoes comfortably	Venous congestion (9), hematoma (1), infection (1), donor site necrosis (1). Total complication rate ∼33%	Not formally measured; authors note cosmetic acceptability and patient satisfaction
Chaput B 2018	Complete necrosis: 2 PPF (6.7%) vs. 1 vsPPF (3.3%) (NS). Coverage failure: 16.7% PPF vs. 3.3% vsPPF	Venous congestion significantly higher in PPF (36.7%) vs. vsPPF (6.7%), *p* = 0.010	Major edema and congestion frequent in PPF; reduced in vsPPF, fewer sutures had to be released	All successful flaps provided stable coverage; vsPPF shortened hospital stay (7.1 vs. 8.8 days, *p* = 0.026). Both groups achieved adequate soft tissue coverage	Distal necrosis: 30% PPF vs. 3.3% vsPPF (*p* = 0.012). Any complication: 36.7% PPF vs. 10% vsPPF (*p* = 0.030). Rescue measures (stitch removal, leeches, heparinization) more frequent in PPF	Not formally measured; cosmetic/functional satisfaction implied
Özalp B 2016	All flaps survived completely. No partial or total necrosis. One patient died of disseminated intravascular coagulation unrelated to flap failure	No venous congestion observed; only transient hyperemia in 2 cases with double perforators	Transient hyperemia (not true venous congestion) in 2 cases	Good reconstruction in 6/6 surviving patients: stable coverage, return to daily activities, good texture and color match; donor site morbidity acceptable (mostly skin grafted)	None in surviving patients (no necrosis, infection, or delayed healing). One unrelated systemic death	Not formally assessed; patient/family satisfaction ∼86%
Cadenelli P 2015	Complete survival; no necrosis or wound dehiscence	Adequate perfusion confirmed intraoperatively; no vascular crisis	Donor site left open temporarily due to edema; closed primarily after 5 days	Stable knee coverage, no recurrence, good local function	None	Not formally assessed
Kang JS 2016	Complete survival, uneventful	Adequate after rotation; no crisis	Mild distal ecchymosis and temporary venous congestion, resolved spontaneously	Stable heel coverage, healed without arterial or venous insufficiency; patient recovered from wound	Only temporary venous congestion; no necrosis	NR
Ademola SA 2015	All flaps survived; 100% skin graft take on donor sites	No venous congestion observed; only transient hyperemia in 2 cases with double perforators	Not described; only seroma and hematoma reported	Stable coverage, complete healing, good color and thickness match	Case 1: seroma drained; grade 2 pressure ulcer (healed conservatively). Case 2: hematoma after external fixator adjustment (resolved after drainage). No necrosis	NR
Horta R 2014	Complete survival, uneventful healing	Preserved; venous supercharging prevented congestion	No significant swelling noted	Stable coverage, full wound healing	None	NR
Cinpolat A 2014	All 7 flaps survived completely	2 patients developed transient distal venous congestion, resolved spontaneously	Mild congestion-related edema only (resolved without sequelae)	All patients ambulated without difficulty within 1 month; excellent esthetic and functional recovery	Only transient venous congestion (2 cases); no necrosis, no donor site morbidity	Not formally reported; cosmetic/functional results considered satisfactory
Dong KX 2014	1–18 months (mean 13.5 months)	One case of venous crisis, resolved after suture release and drainage	Not significant; no persistent engorgement reported	Good texture, color, contour; scars minimal with primary closure; Zhang scale 8–10 in all patients, indicating high satisfaction	1 venous crisis; no necrosis, no ulceration, no major donor site morbidity	Not formally assessed; Zhang scale used for satisfaction
Innocenti M 2014	4/5 survived uneventfully; 1 patient required early above-knee amputation due to life-threatening sepsis, flap still viable at amputation	Preserved, no flap loss due to venous congestion	Some donor sites required skin graft if >6 cm; otherwise closed primarily	Reliable coverage of tibial tuberosity and patella; muscle covered tibia/dead space, propeller skin covered patella/distal femur; stable coverage, pliable skin	1 systemic sepsis leading to amputation; no local flap complications	Not formally assessed; clinical satisfaction reported
Chang SM 2014	All flaps survived	No significant venous congestion	Mild swelling in 2 cases, resolved with conservative measures	Functional recovery (good walking ability, partial range of motion restored)	Mild venous congestion (2 cases), superficial infection (1 case)	Not formally assessed, implied satisfaction
Tos P 2011	26/28 flaps survived; 2 partial necrosis with successful treatment	No venous compromise or congestion observed	Mild transient swelling in 2 cases, resolved without intervention	All patients achieved functional recovery (walking, restored sensation) and cosmetic outcomes were satisfactory	28.6% complication rate; partial flap necrosis in 10.7%, wound dehiscence in 3.5%, venous congestion in 7.1%	Not formally measured, but functional outcomes and aesthetic satisfaction implied high satisfaction
Rubino C 2009	Flap fully survived; complete wound closure	Not specifically reported; flap remained viable without signs of congestion	Minimal, not reported as clinically significant	Patient achieved stable soft tissue coverage; osteomyelitis controlled; limb function preserved	NR	NR
Pignatti M 2008	All flaps survived; 1 small superficial tip necrosis due to scar inclusion	Two cases of transient venous congestion, resolved spontaneously	Not significant; only transient congestion in 2 cases	Complete and stable soft tissue coverage; donor site inconspicuous; good aesthetic outcome; all patients regained functionality appropriate to limb	1 superficial tip necrosis; transient venous congestion in 2 cases	Not formally assessed; implied satisfaction via functional and aesthetic results
Moscatiello F 2007	No total flap losses; 1 partial necrosis >20% in a diabetic patient	NR	NR	Stable coverage of knee and upper leg defects; flap reliable in all cases	Partial necrosis >20% in 1 diabetic patient; otherwise none	NR

Studies originated from 21 countries, mainly China (29.4%), Italy (11.8%), and India and Turkey (7.8% each), with smaller contributions from Europe, Asia, Africa, and the Americas, reflecting wide but uneven global representation.

Across studies reporting sex (*n* = 49), the pooled population included 1474 participants—66% male and 34% female—showing a general male predominance.

Regarding design, case series prevailed (49.0%), followed by cohort studies (33.3%) and case reports (15.7%). Most were observational and retrospective. Follow-up data were available in 44 studies, with a median duration of 12 months (IQR 6–25), extending up to 84 months in a few long-term studies.

### Etiology–location profile and flap configuration

#### Etiology of defects

**Figure 2:**
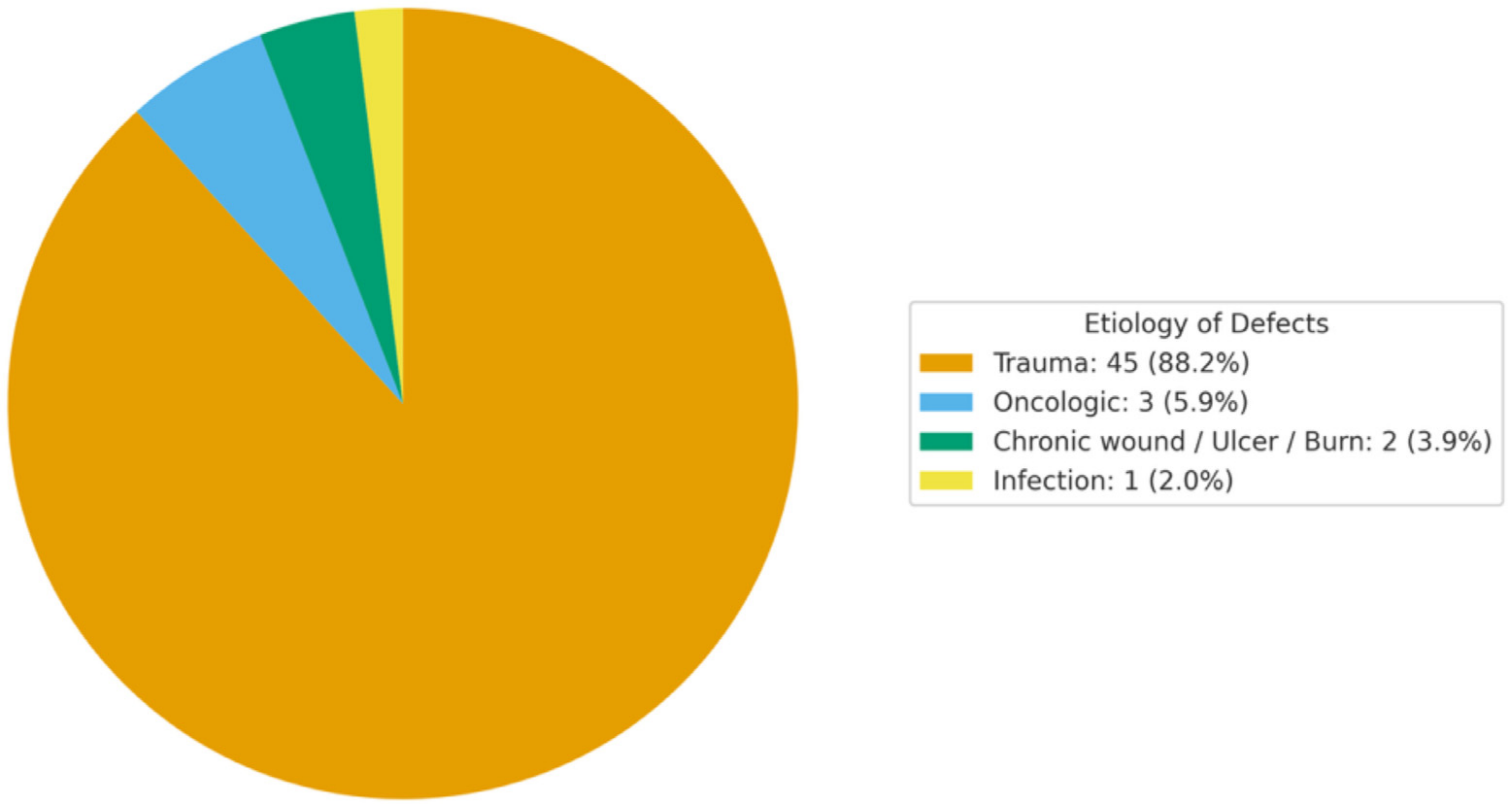
Pie chart reporting findings on etiology of defects.

As reported in Figure 2 (pie chart), most studies addressed trauma-related defects (45/51; 88.2%), followed by oncologic resections (3/51; 5.9%), chronic wounds/ulcers or burns (2/51; 3.9%), and a minority investigating infectious causes (1/51; 2.0%). This distribution highlights the predominant role of traumatic etiologies in the included series, with limited contributions from oncologic, chronic wound, or infectious contexts.

#### Defect locations

**Figure 3:**
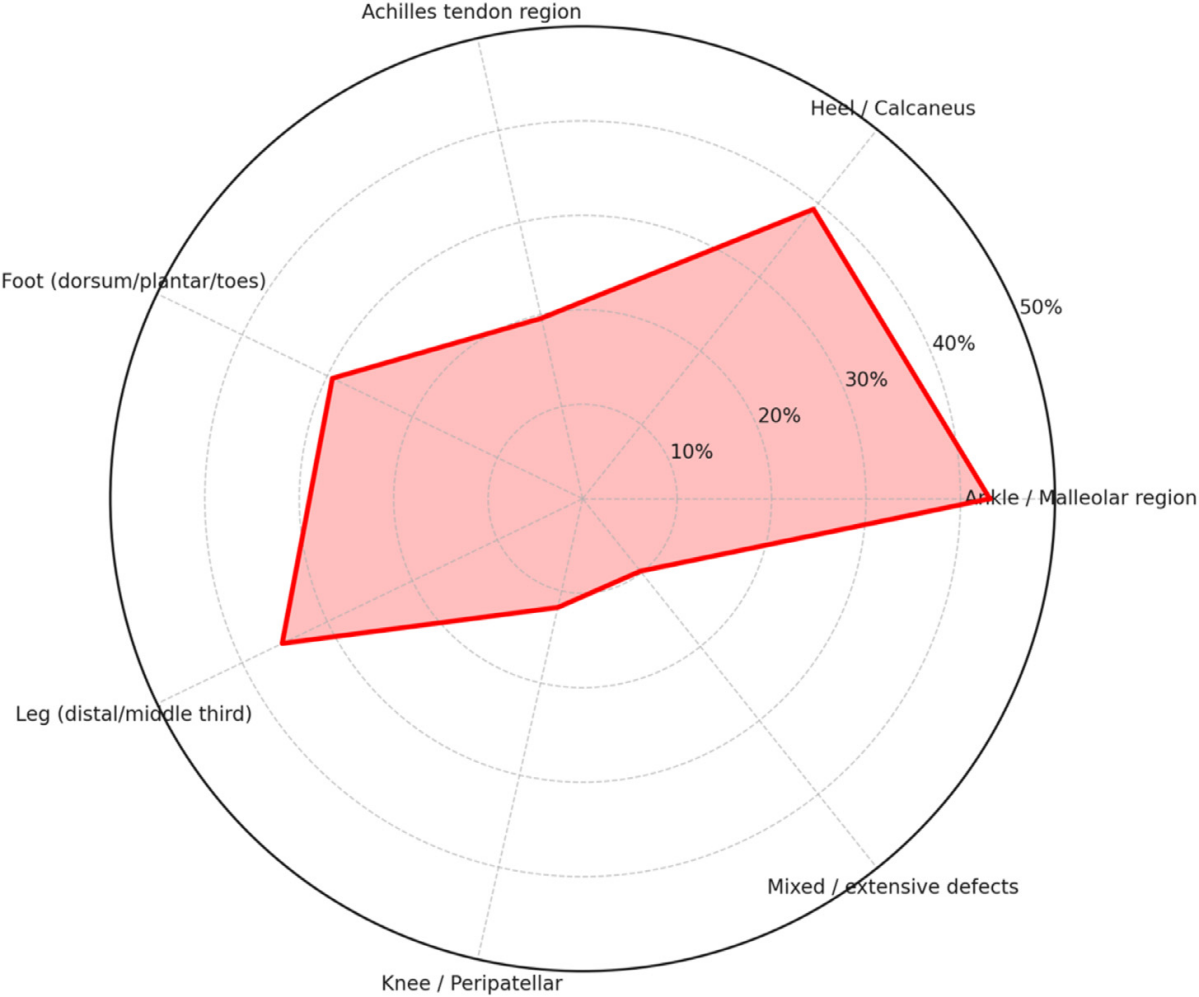
Radar chart reporting findings on defects locations.

As reported in Figure 3 (radar chart), most reconstructions involved the ankle/malleolar region (22/51; 43.1%) and the heel/calcaneus (20/51; 39.2%). Leg defects—mainly distal/middle third with exposed bone/plates/tendon—accounted for 18/51 (35.3%), and foot defects (dorsum/plantar/toes) for 15/51 (29.4%). The Achilles tendon region was reported in 10/51 (19.6%) cases, frequently associated with heel or distal leg wounds. Knee/peripatellar lesions were less common (6/51; 11.8%), while mixed/extensive lower-limb defects spanning multiple subunits occurred in 5/51 (9.8%).

#### Flap types

**Figure 4:**
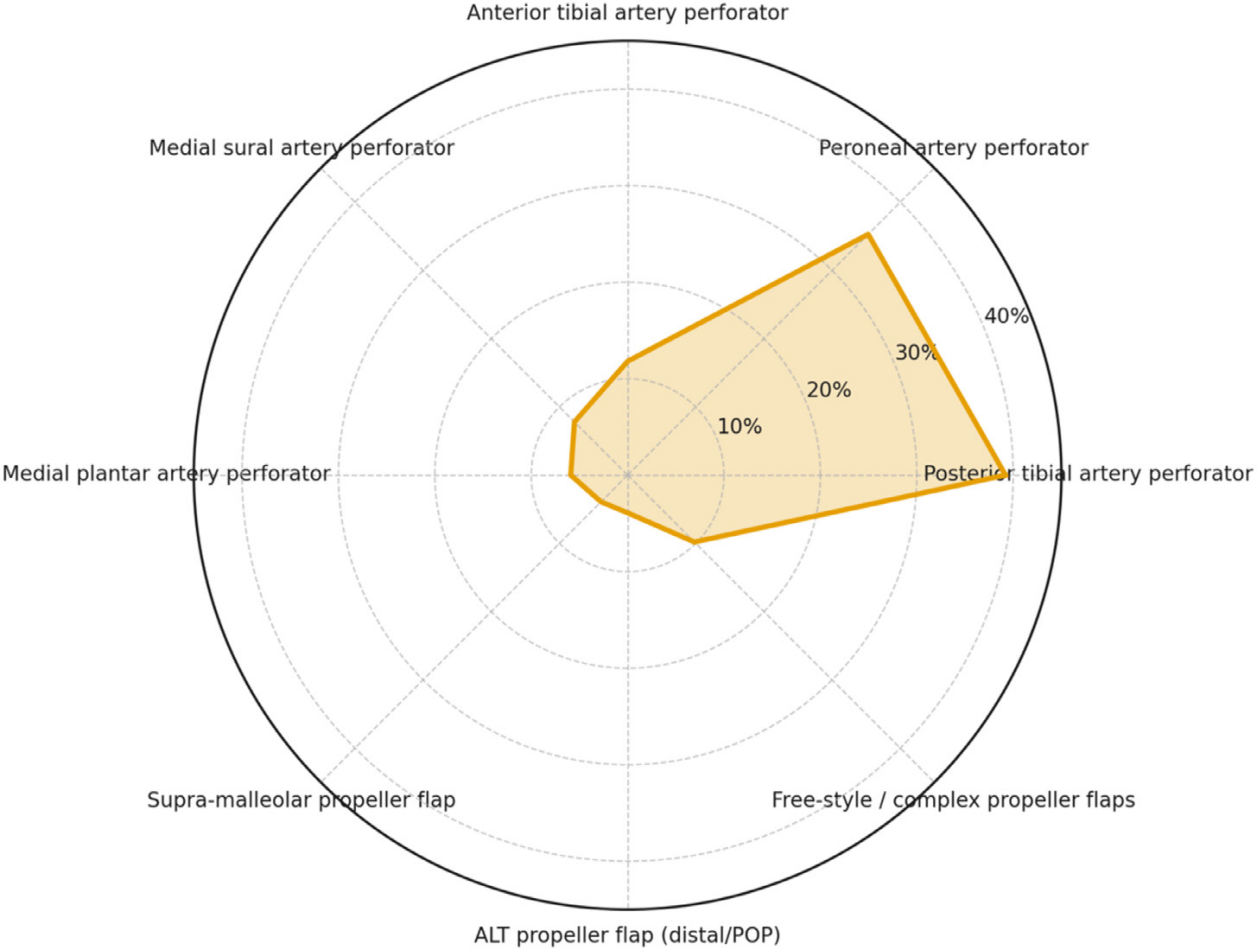
Radar chart reporting findings on flap types.

As reported in Figure 4 (radar chart), all included studies employed PPFs, with the posterior tibial artery (39.2%) and peroneal artery (35.3%) representing the predominant vascular sources. Less frequent donor sites included the anterior tibial artery (11.8%), medial sural artery (7.8%), and medial plantar artery (5.9%). Rarely reported were supra-malleolar flaps (3.9%), distal ALT propeller flaps (3.9%), and free-style or complex propeller designs (9.8%). This distribution highlights the central role of tibial and peroneal perforators in propeller-based reconstructions.

### Outcomes

#### Flap survival

**Figure 5:**
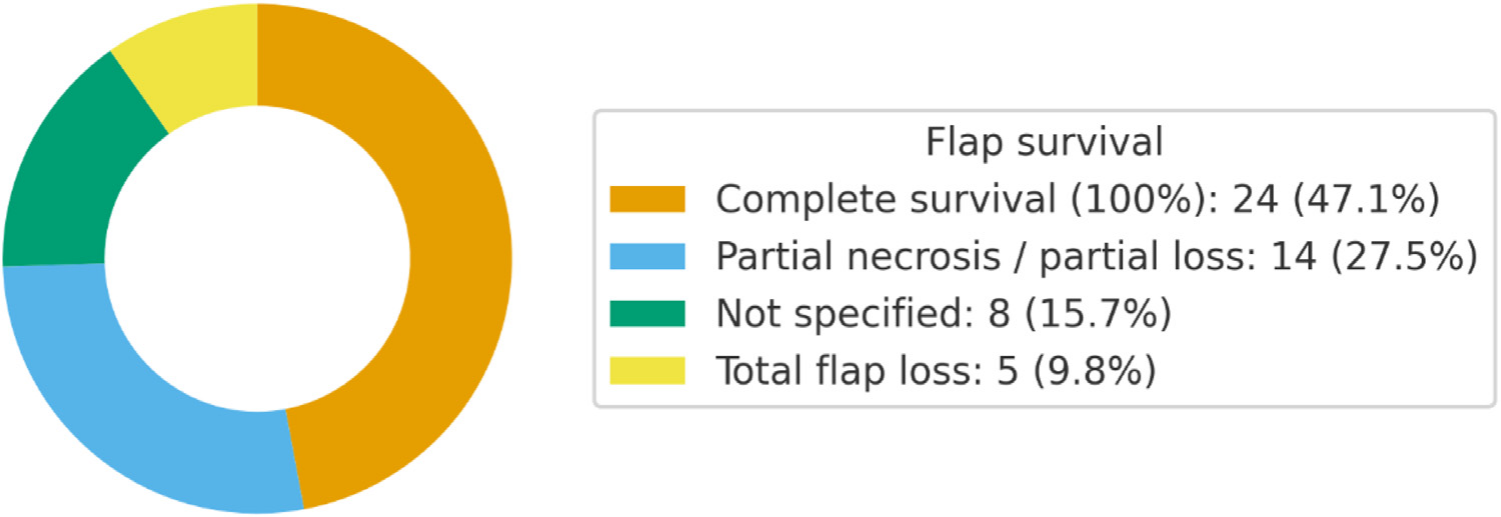
Donut chart reporting findings on flap survival.

Complete flap survival was reported in 24 studies (47.1%), while partial necrosis or partial loss occurred in 14 studies (27.5%). Total flap loss was documented in 5 studies (9.8%), whereas in 8 studies (15.7%) survival was not specified. These findings, illustrated in Figure 5 (donut chart), highlight an overall high flap survival rate, with partial or total losses representing a minority of cases.

#### Flap swelling

Reporting on flap swelling was heterogeneous. Most studies did not specify swelling outcomes (31/51; 60.8%). Swelling without severity grading was noted in 9 studies (17.6%), while mild or transient swelling was described in 7 studies (13.7%) and severe or persistent swelling in 4 studies (7.8%). Notably, one study provided a detailed grading, reporting flap swelling in 6 grade I and 9 grade II cases at 7 days, which decreased to 13 grade I and 2 grade II cases at 6 months.

#### Venous patency

**Figure 6:**
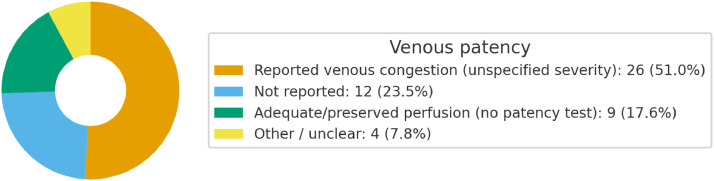
Donut chart reporting findings on venous patency.

After recategorization into more informative groups, venous congestion (unspecified severity) was reported in 26/51 studies (51.0%). Not reported accounted for 12/51 (23.5%), while adequate/preserved perfusion without a formal patency test was described in 9/51 (17.6%). The remaining 4/51 (7.8%) were other/unclear descriptions. These proportions are shown in Figure 6 (donut chart), highlighting that congestion-related issues were the most commonly documented venous events, whereas formal assessments of venous patency were infrequently reported.

#### Functional outcomes

**Figure 7:**
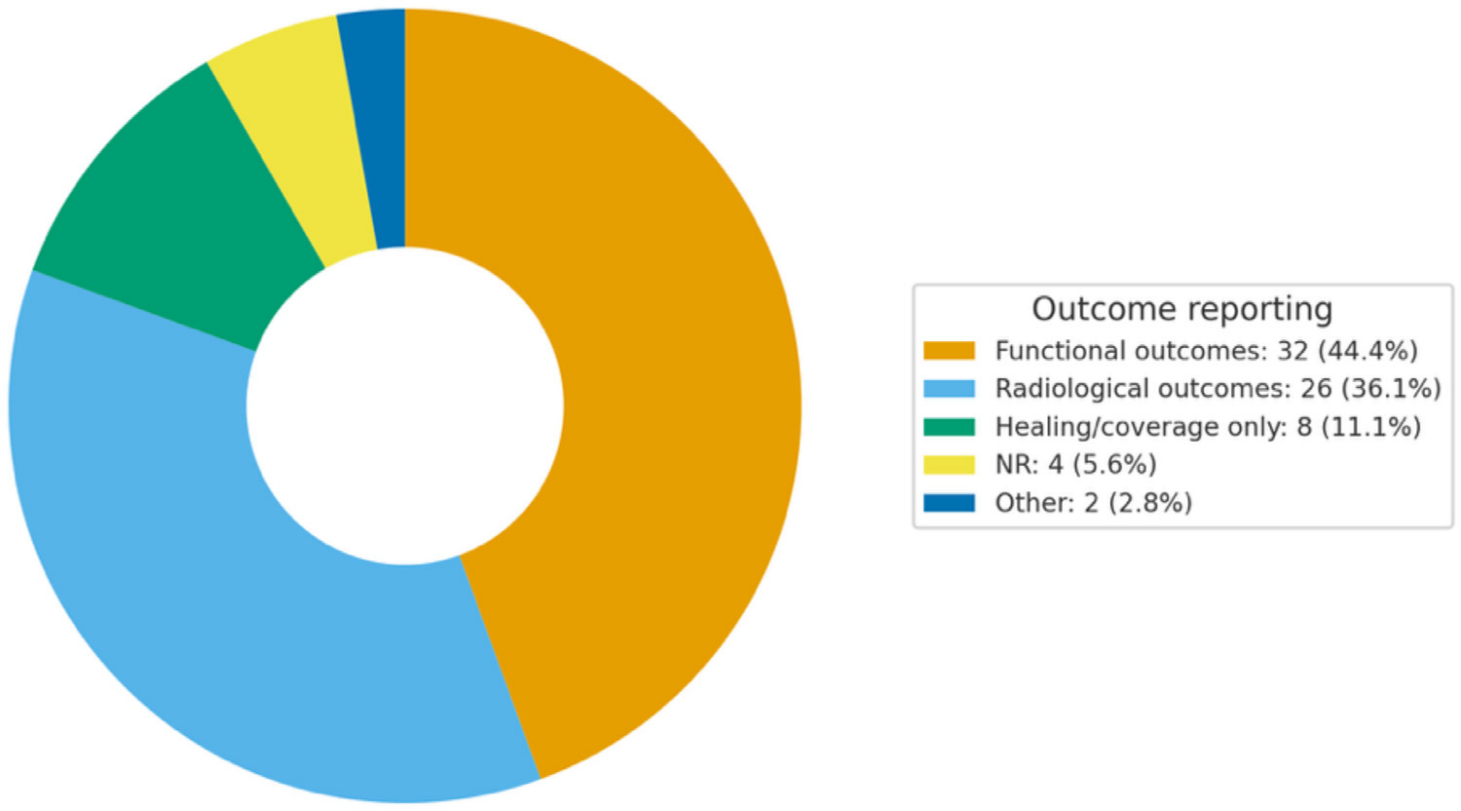
Donut chart reporting findings on clinical outcomes distribution.

As illustrated in Figure 7 (donut chart), outcome reporting was heterogeneous, and studies could contribute to more than one category. Functional outcomes were reported in 32 counts (44.4%), including validated scoring systems such as AOFAS (American Orthopedic Foot & Ankle Society), MSTS (Musculoskeletal Tumor Society), LEFS (Lower Extremity Functional Scale), or FAAM (Foot and Ankle Ability Measure), as well as descriptions of gait recovery, return to work, or range of motion. Radiological outcomes were described in 26 cases (36.1%), primarily focusing on bone union, consolidation, callus formation, RUST scoring, or the absence of osteomyelitis, which was sometimes confirmed by radiographs, CT scans, or MRI. Healing/coverage only was reported in 8 counts (11.1%), limited to descriptions of stable soft-tissue coverage or wound healing without functional or imaging assessment. Not reported (NR) applied to 4 counts (5.6%), where outcomes were explicitly absent, while Other (2 counts, 2.8%) included non-standardized or unclear descriptions.

#### Complications

**Figure 8:**
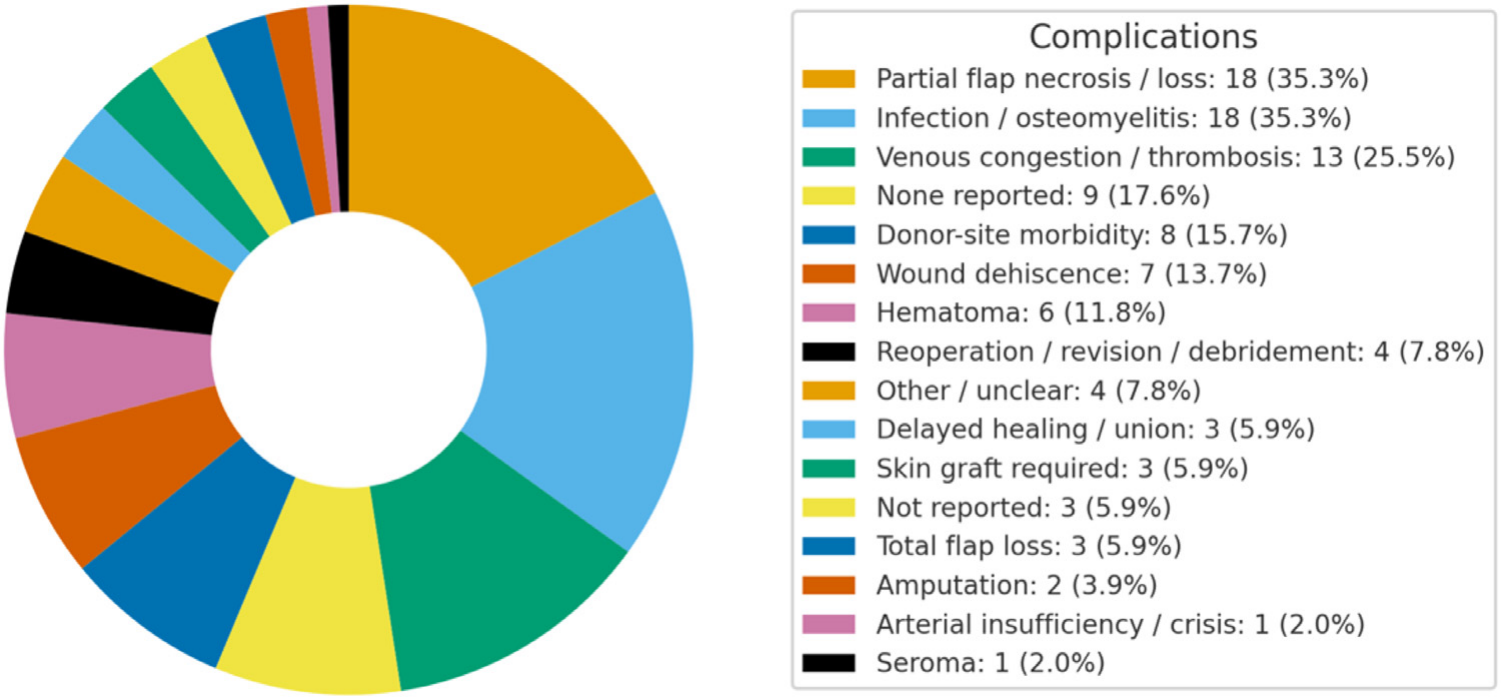
Donut chart reporting findings on complications.

Across the 51 studies, any complication was reported in 44/51 (86.3%), none in 4/51 (7.8%), and not reported in 3/51 (5.9%) (Figure 8). At the event/category level (multi-label, as a study can contribute >1 event), the most frequent findings were infection/osteomyelitis (18; 35.3%) and partial flap necrosis/loss (18; 35.3%), followed by venous congestion/thrombosis (13; 25.5%). Additional complications included donor-site morbidity (8; 15.7%), wound dehiscence (7; 13.7%), hematoma (6; 11.8%), reoperation/revision/debridement (4; 7.8%), delayed healing/union (3; 5.9%), skin graft required (3; 5.9%), total flap loss (3; 5.9%), amputation (2; 3.9%), arterial insufficiency/crisis (1; 2.0%), and seroma (1; 2.0%). A residual Other/unclear group (4; 7.8%) captures ambiguous statements that mixed vague descriptors with necrosis terminology and could not be unambiguously reassigned.

#### PROM (patient-reported outcome measure)

**Figure 9:**
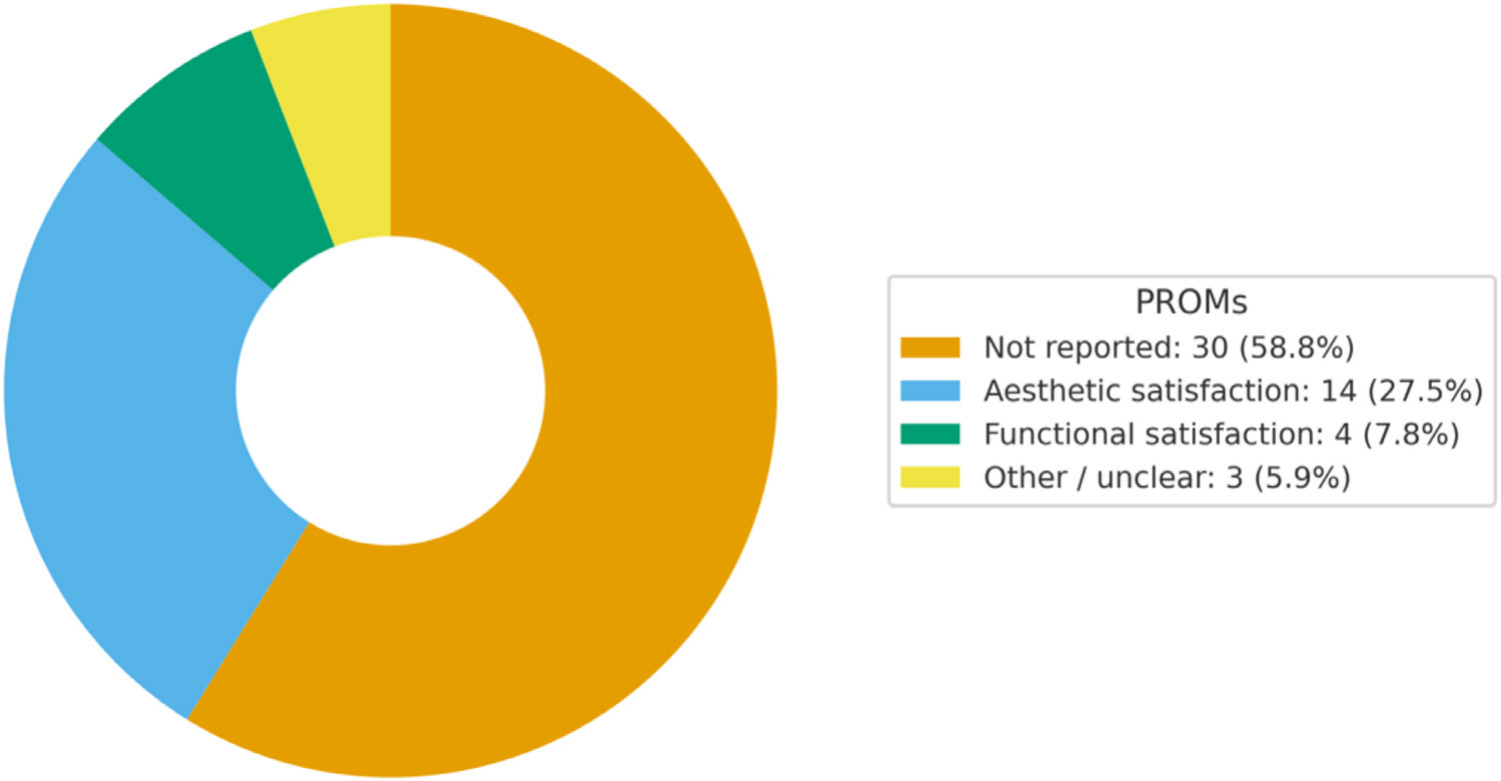
Donut chart reporting findings on PROMs.

PROMs were inconsistently assessed across the included studies (Figure 9). In most cases, they were not reported (30/51; 58.8%). Aesthetic satisfaction was described in 14/51 studies (27.5%), usually through qualitative or non-validated assessments. Functional satisfaction was reported in 4/51 studies (7.8%), and this group also included the only standardized PROM identified, the LIMB-Q™, a validated questionnaire specifically designed for lower-limb reconstruction that measures function, appearance, and quality of life.^56^ A small fraction (3/51; 5.9%) provided unclear or ambiguous information. Overall, validated PROM use was extremely rare, with most studies relying on cosmetic satisfaction or non-standardized assessments rather than structured patient-reported instruments.

### Main findings

Overall, across the 51 studies, PPFs were consistently reported as reliable with high flap survival (dominant theme), although partial/distal necrosis was a recurring complication. Venous congestion—often mitigated by distal venous “supercharging” or adjunct anastomoses—was commonly discussed. Functional outcomes were frequently favorable when assessed, and several reports documented radiographic bone union without osteomyelitis in tibial/ankle reconstructions. Donor-site morbidity was generally low with frequent primary closure. Anatomically, the technique was highlighted as effective for covering the heel/Achilles and ankle/malleolar areas. Some comparative or mixed-technique series have noted that propeller flaps can reduce the need for free flaps in selected defects. Meanwhile, pre-operative planning tools (CTA/ICG/Doppler/3D) and attention to rotation arcs (up to ∼180°) are emphasized to optimize perfusion. Advanced strategies, such as relay/sequential propeller flaps, and, in complex trauma, combinations with external fixation/Ilizarov, were described to expand indications and manage donor sites.

### Risk of bias assessment

**Figure 10:**
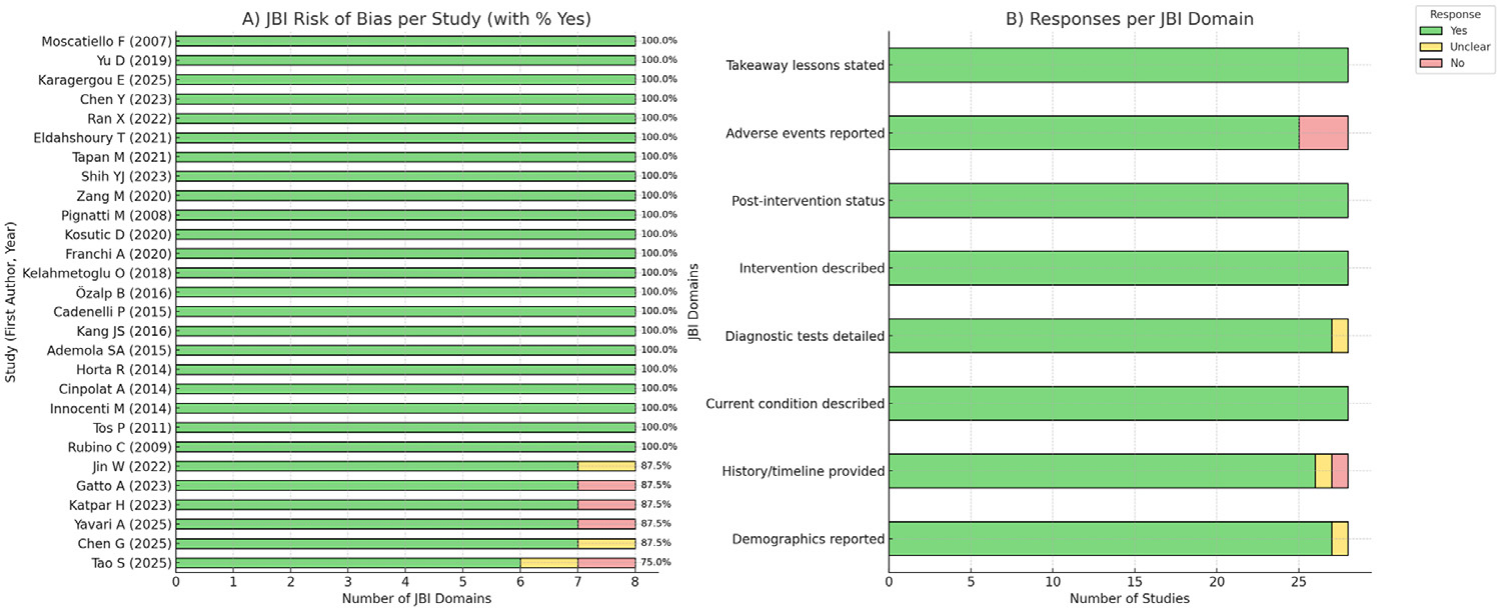
JBI risk of bias across studies (Panel A) and across domains (Panel B).

**Figure 11:**
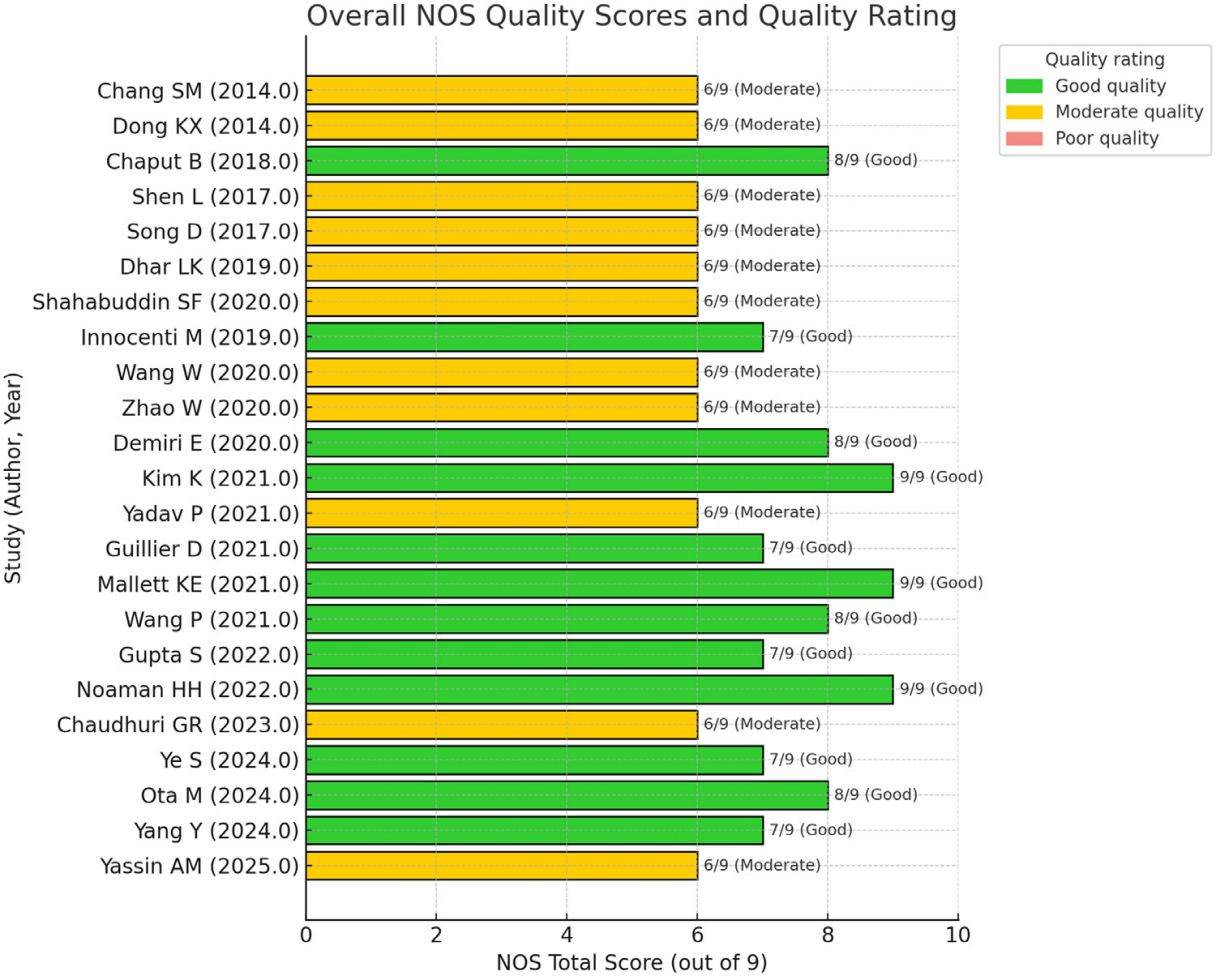
Risk of bias assessment according to the Newcastle Ottawa Scale (NOS).

A total of 28 studies, including case reports, case series, and comparative designs, were assessed using the Joanna Briggs Institute (JBI) and Newcastle–Ottawa Scale (NOS) tools (Figure 10, Figure 11). Overall methodological quality was good, with most descriptive studies fulfilling 6–8 of 8 JBI criteria (average compliance >85%) and none rated at high risk of bias (Figure 10). Stronger areas included reporting of demographics, interventions, and outcomes, while adverse events and clinical implications were less consistently described. Among cohort and comparative studies (*n* = 23), NOS scores ranged from 6 to 9 (median = 8), with most classified as “Good” and none as “Poor” (Figure 11). Overall, the evidence was methodologically robust and at low risk of bias, despite limitations from retrospective, non-randomized designs.

## Discussion

The main findings of this systematic review suggest that PPFs represent a technically feasible, but not universally predictable, option for the reconstruction of soft-tissue defects of the distal lower limb. Across the included studies, flap survival was generally satisfactory; however, complication rates varied considerably, and functional recovery was inconsistently assessed. The evidence, largely descriptive and retrospective, does not yet allow firm conclusions on comparative efficacy or long-term outcomes.

Although overall survival rates were acceptable, partial necrosis, venous congestion, and delayed healing were common events, occurring in up to one-third of cases. These complications have been repeatedly reported as the main limitations of propeller flap reconstruction, particularly in the distal third of the leg where venous outflow is often precarious.^57^, ^58^, ^59^ The predominance of trauma-related defects (≈88%) may partly explain the high incidence of venous congestion, since soft-tissue edema and local vascular compromise are typical of these settings.^60^

The functional and patient-reported outcomes reported across studies remain poorly standardized. Only a few authors used validated tools such as AOFAS, MSTS, or LEFS scores, while most relied on descriptive terms such as “satisfactory gait” or “return to activity.” Previous meta-analyses have highlighted that the absence of standardized functional measures is one of the main weaknesses of current evidence on PPFs.^38^ Furthermore, follow-up periods were frequently short and heterogeneous, which limits the assessment of durability, recurrence, or donor-site morbidity.

Technical refinements, including venous supercharging or the use of sequential (relay) flaps, have been proposed to mitigate congestion and enhance flap reliability.^5^^,^^43^^,^^60^ Chaput et al.^43^ prospectively compared conventional and venous-supercharged propeller flaps, reporting lower rates of congestion in the latter, but these findings were based on small cohorts. Similarly, Mishra et al.^5^ reported promising results with supercharged PPFs; however, the added technical complexity and lack of standardized comparison make generalization difficult. These limited data suggest potential benefit, but not definitive proof, of adjunctive venous augmentation.

Propeller flap reliability is also influenced by flap size and design parameters, although these were inconsistently reported across the included studies and rarely allowed a quantitative synthesis. In clinical practice, perfusion constraints related to the perforasome concept, perforator caliber/quality, and the distance between the selected perforator and the defect may limit the safe dimensions of the skin paddle, particularly in the distal third of the leg where vascular reserve and venous outflow can be precarious. Additional factors such as the arc of rotation (up to 180°), pedicle torsion, flap thickness, and tension at inset may further affect distal perfusion and venous drainage. When reported, technical planning aimed at selecting a reliable perforator and optimizing flap geometry (including perforator-to-defect distance) was considered important to reduce the risk of congestion and partial necrosis, but the available literature does not support universal cut-offs. Overall, standardized reporting of defect size, flap dimensions, and objective intraoperative perfusion assessment would be essential to better define size-related safety thresholds in future studies.

When compared with free perforator flaps, PPFs appear less demanding and more cost-effective, but not necessarily superior in outcomes. Innocenti et al.^5^ found similar complication rates between the two techniques in a large comparative series, although propeller flaps were associated with shorter operative times. These results support their use in selected cases, but not as a universal replacement for microsurgical reconstruction.

With respect to contraindications and patient selection, the included studies variably described comorbidities and local wound factors, limiting firm conclusions; however, several clinically relevant considerations emerge. Propeller flaps may be less suitable—or require heightened caution—in the presence of compromised local vascularity (e.g., severe peripheral arterial disease or unreliable perforators), extensive scarring/previous surgery within the perforator territory, significant oedema within the trauma zone, and an unstable or infected wound bed when adequate debridement and optimization are not achievable. Patient-related risk profiles such as poorly controlled diabetes, heavy smoking, and significant systemic comorbidity may also increase the likelihood of venous congestion and partial necrosis. Therefore, careful patient selection, meticulous perforator choice and dissection, and appropriate preoperative planning (e.g., Doppler or CTA in selected cases) remain key to minimize complications and optimize outcomes.

From a methodological standpoint, the overall quality of available studies remains modest. Most descriptive papers met 6–8 JBI criteria yet often lacked details on adverse events or follow-up completeness. Cohort studies scored 6–9 on the NOS, indicating moderate-to-good quality but persistent heterogeneity. The literature on PPFs is largely based on small, single-center, retrospective studies, limiting validity and reproducibility, and the lack of randomized data warrants cautious interpretation.

Despite these limitations, PPFs offer clear practical advantages: they can be performed under regional anesthesia, avoid microsurgical anastomosis, and preserve major vascular trunks—benefits particularly relevant for comorbid or high-risk patients. Advances in preoperative imaging, such as CT angiography and indocyanine green mapping, may enhance flap selection and reduce complications, though confirmatory evidence remains limited.

### Limitations and strengths

This review shares the limitations of the available evidence. Included studies were heterogeneous in design, population, and outcomes, mostly retrospective case series with small samples and limited follow-up, making publication bias possible. Functional and patient-reported outcomes were rarely assessed, and the lack of standardized definitions of complications (e.g., “partial necrosis,” “flap loss”) limited comparisons. In addition, defect size and flap dimensions, as well as explicit contraindications/patient selection criteria, were inconsistently reported, preventing a robust synthesis of size-related thresholds and clinical selection guidance.

Strengths include the systematic methodology and use of validated tools (JBI and NOS), enabling transparent quality appraisal and synthesis of descriptive and comparative data. The inclusion of over 1400 patients across 51 studies offers a broad overview of current practice, though inference strength remains limited.

Operative time was inconsistently reported across the included studies and, when available, showed marked heterogeneity in definitions and surgical settings. As a result, quantitative synthesis was not feasible. Nevertheless, shorter operative times were occasionally reported for propeller perforator flaps compared with free flap reconstruction. This observation underscores the need for more standardized reporting of intraoperative parameters in future studies.

## Conclusion

In summary, PPFs represent a useful option for lower-limb reconstruction but show variable reliability and inconsistent functional outcomes. Current evidence confirms satisfactory coverage in selected cases, yet complication rates—particularly venous issues and partial necrosis—remain significant. Robust, prospective multicenter studies with standardized functional and patient-reported outcomes are required to define their definitive role and optimize surgical indications.

## Funding

Published with a contribution from 5 x 1000 IRPEF funds in favourof the University of Foggia, in memory of Gianluca Montel.

## Data availability statement

All data analyzed in this study are included in the published articles referenced.

## Declaration of competing interest

None of the authors has a financial or other interest to declare.
